# Land use controls the spatiotemporal patterns of surface water quality of the Quadrilátero Ferrífero mineral province, Brazil

**DOI:** 10.1007/s10653-026-03358-7

**Published:** 2026-07-20

**Authors:** Gabriel Soares de Almeida, Rafael Tarantino Amarante, Normara Yane Mar da Costa Andrade, Roberto Dall’Agnol, Prafulla Kumar Sahoo, Paulo Rógenes Monteiro Pontes, Emmanoel Vieira Silva-Filho, Eduardo Duarte Marques, Raquel Fernandes Mendonça, Abraão Gomes Soares Junior, Gabriel Negreiros Salomão

**Affiliations:** 1 Instituto Tecnológico Vale (ITV), Rua Boaventura da Silva, 955, Belém, PA Brasil; 2 Geosciences Institute, Universidade Federal Do Pará, Belém, Pará Brasil; 3 Departament of Environmental Science and Technology, School of Environment and Earth Sciences, Central University of Punjab, VPO-Ghudda, Bathinda, Punjab 151401 India; 4 Programa de Pós-Graduação Em Geociências (Geoquímica), Instituto de Química, Universidade Federal Fluminense, Outeiro São João Batista S/N - Centro, Niterói, RJ 24020-141 Brasil; 5 Serviço Geológico Do Brasil (SGB/CPRM), Avenida Brasil, 1731, Funcionários, Belo Horizonte, Minas Gerais Brasil; 6 Vale S.A., Environmental Management Executive Department, Nova Lima, Minas Gerais Brasil

**Keywords:** Hydrogeochemistry, Water Quality Index (WQI), Land use change, Anthropogenic impacts, Environmental monitoring, Quadrilátero Ferrífero

## Abstract

Surface water quality in the Quadrilátero Ferrífero, a mining province in southeastern Brazil, was evaluated by combining water quality indices (WQI), land use and land cover (LULC), and population data to identify spatial and seasonal influences on river chemistry. We analyzed 835 (rainy) and 845 (dry) samples and calculated WQI scores using established methods from the literature. The methods for calculating the WQI detected broad signals of eutrophication and those related to sanitation but were less sensitive to inorganic and potentially toxic elements (PTE), whereas CCME, especially when including PTE variables, offered a more rigorous and adaptable assessment for mining-influenced areas. Microcatchments were classified into LULC groups based on the proportional dominance and co-occurrence of classes. Across methods and seasons, WQI values showed a clear degradation gradient aligned with increasing human activity. Catchments dominated by natural cover and low-intensity uses consistently exhibited the highest WQI values; notably, mining-influenced classes with substantial natural vegetation often clustered towards this higher-quality end. In contrast, catchments shaped by human occupation in urban and rural settings showed the lowest WQI scores and were statistically distinct from the low-intensity group in most pairwise comparisons, although overlaps occurred among the anthropogenic classes depending on method and season. Total population per micro-catchment was inversely related to WQI results by IGAM method in both seasons, highlighting the impact of urban expansion and infrastructure pressures on water-quality decline. Overall, the LULC-based microcatchment classification was crucial for understanding how dominant land use patterns influence water quality and for guiding targeted watershed management in mining regions.

**Supplementary Information:**

The online version contains supplementary material available at 10.1007/s10653-026-03358-7.

## Introduction

The Quadrilátero Ferrífero (QF) mining province, located in Minas Gerais, southeastern Brazil, is internationally recognized for its exceptional mineral endowment. The region hosts one of the world’s largest iron ore reserves, in addition to economically important deposits of manganese, gold, bauxite, and ornamental stones, making it a strategic center for mineral extraction and processing (Vicq et al., 2015; Weber et al., 2017). However, intensive mining activities have led to a rapid increase in urban occupation, which, combined, have caused rapid land use and land cover (LULC) changes, exerting increasing pressure on surface water systems, with potential consequences for water quality and availability.

Rivers play a fundamental role in sustaining ecosystems and supporting human activities, making water quality a central concern for environmental management and public health. Surface water quality in the QF has been recurrently affected by the cumulative impacts of economic activities, particularly mining, agriculture, and urban expansion (Almeida et al., 2025; Medeiros Filho et al., 2025). Agricultural practices contribute nutrient loads, primarily nitrogen- and phosphorus-based compounds, promoting eutrophication and altering the physicochemical balance of surface waters. In parallel, deforestation and soil sealing associated with land conversion reduce infiltration capacity, intensify surface runoff, and enhance erosion processes, leading to increased turbidity and total suspended solids. Unplanned urban growth, often characterized by inadequate solid waste management and insufficient sewage infrastructure, further aggravates water contamination in the region (Chapman, 2021; Chidiac et al., 2023; Shil et al., 2019; Toledo & Nicolella, 2002; Uddin et al., 2022).

Hydrological processes play a fundamental role in controlling the transport and fate of pollutants within watersheds. During the rainy season, increased surface runoff promotes the mobilization of sediments, nutrients, organic matter, and potentially toxic elements accumulated in urban, agricultural, and mining areas, facilitating their transfer to river systems (Allan, 2004; Giri & Qiu, 2016). In addition to runoff, erosion processes and the hydrological connectivity between terrestrial and aquatic environments may intensify the delivery of contaminants to watercourses, contributing to seasonal variations in water quality (Bracken et al., 2013; Covino, 2017). Conversely, during dry periods, the reduced influence of runoff and the greater contribution of groundwater inputs generally favor more stable hydrochemical conditions (Allan, 2004; Giri & Qiu, 2016; Shil et al., 2019).

Assessing surface water quality in the QF is therefore essential from both environmental and socioeconomic perspectives. The region supports approximately 7.7 million inhabitants who depend on river systems for drinking water supply, irrigation, and industrial use. Degradation of these water bodies compromises aquatic biodiversity and disrupts key ecosystem services, including water purification, climate regulation, agricultural productivity, and public water supply. Although several studies have addressed water contamination in parts of the QF (Dias & Gonçalves, 2024; Fraga et al., 2021; Medeiros et al., 2012; Parra et al., 2007; Piazi et al., 2018), most investigations remain spatially fragmented or focused on individual basins, leaving critical knowledge gaps regarding regional-scale patterns, pollutant dispersion mechanisms, and the cumulative effects of multiple anthropogenic pressures.

In this context, the Water Quality Index (WQI) has been widely adopted as an integrative tool that synthesizes multiple physical, chemical, and biological parameters into a single numerical value, facilitating the assessment of the conservation status of water bodies. The WQI enables the identification of spatial and temporal trends, supports comparisons among different watersheds, and provides a scientifically robust basis for environmental planning and decision-making (Behmel et al., 2016; Chapman, 2021; Chidiac et al., 2023).

The applicability of the WQI has been demonstrated across a wide range of spatial scales, from small catchments to large international river systems, highlighting its versatility for water quality assessment under diverse environmental conditions (Toledo & Nicolella, 2002; Khan et al., 2003; Lumb et al., 2006; Marques et al., 2007; Javed et al., 2017; Shil et al., 2019; Uddin et al., 2022). Importantly, the use of different WQI formulations within the same study area allows for a more comprehensive evaluation of water quality, as methodological differences influence parameter sensitivity and classification thresholds. This comparative approach enhances the interpretation of pollution sources and supports the prioritization of critical areas for management and remediation, particularly in regions subject to complex and overlapping anthropogenic impacts (Behmel et al., 2016; Chidiac et al., 2023; Uddin et al., 2022).

Within this framework, an integrated regional assessment of surface water quality in the QF, combining multiple WQI methodologies and LULC analysis, is crucial to improving the understanding of hydrogeochemical processes and anthropogenic controls on water quality. Such an approach provides valuable insights for environmental governance, supports evidence-based public policies, and contributes to the sustainable management of water resources in one of Brazil’s most economically and environmentally strategic mining provinces (Bressane et al., 2023).

This study aims to address these gaps by (1) analyzing the spatial and temporal patterns of surface water quality, (2) identifying key pollution sources and the influence of different LULC types, and (3) evaluating the effectiveness of various WQI methodologies as management tools. The findings are intended to provide a scientific basis for informed public policies and sustainable water resource management in this region of intense mining activity.

## Study area

**Fig. 1:**
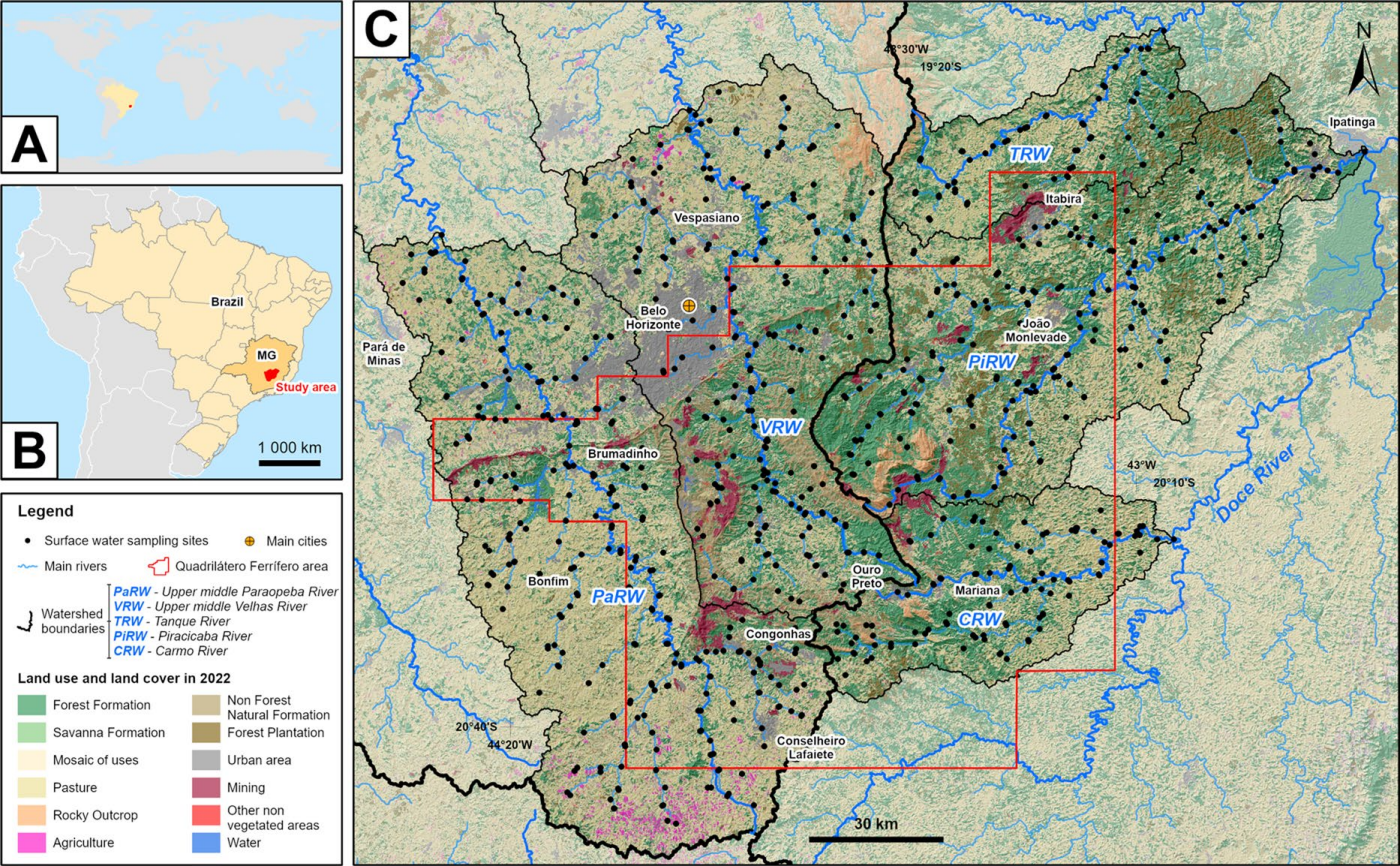
Location of the study area showing the Quadrilátero Ferrífero, Minas Gerais State, Brazil. The main cities and the distribution of land use and land cover, highlighting mining, urban, agricultural, forested, pasture, and rock outcrop areas. The map also shows the main river basins that divide QF and the sampling points distributed along its drainage network. Source: Almeida et al. (2025)

Although the QF covers approximately 7000 km^2^ (delineated by the red boundary in Fig. 1), the survey conducted in this study for calculating and analyzing the WQI encompassed a significantly larger hydrographic area, on the order of ~ 25,000 km^2^ (also shown in Fig. 1). Its rugged topography features elevations ranging from 600 to 1,800 m and a tropical highland climate characterized by distinct rainy summers and dry winters (Alvares et al., 2013). The region supports diverse vegetation, with remnants of the Atlantic Forest at higher altitudes and Cerrado formations in the intermontane valleys (Instituto Brasileiro de Geografia e Estatistica—IBGE, 2006). The soils are predominantly Latosols developed on granitic-gneissic bedrock, with Litholic Neosols on mineralized outcrops, Cambisols associated with metamafic rocks, and scattered Argisols (Fundação Estadual do Meio Ambiente—FEAM, 2007).

The QF exhibits a dense and complex drainage network. The main watercourses belong to the Doce River basin to the east, including the Piracicaba, and Carmo rivers, and the São Francisco River basin to the west, featuring the Paraopeba and Velhas rivers (Fig. 1). These watersheds are vital for regional water supply, supporting urban populations, agricultural irrigation and industrial activities.

Regarding the LULC coverage of the study area (Fig. 1), data from MapBiomas Project (2026) reveals a landscape markedly shaped by anthropogenic activities, where mining is the predominant economic activity in QF, followed by agriculture and livestock farming. Agricultural areas, including poultry farming, cattle and swine raising, and the cultivation of sugarcane, soybean, corn, beans, and coffee, occupy approximately 43.5% of the territory. Despite occupying a relatively small area (< 1%), the mining sector exerts a disproportionately large environmental and socioeconomic impact. Its operations extract a range of resources, primarily iron ore, gold, and manganese, alongside construction aggregates (sand, gravel) and gemstones (emerald, topaz). Native vegetation, composed of remnants of tropical forests and savanna formations (Cerrado), still accounts for a significant portion of the land cover, encompassing approximately 44.8% of the region. These ecosystems play a critical role in maintaining essential ecological functions, such as hydrological regulation and biodiversity conservation. Other land uses include silviculture (3.9%, predominantly eucalyptus and pine), urban areas (5.2%), rock outcrops (1.2%) and water bodies (0.4%). The industrial sector, especially the steel and metallurgical industries, is also an important component of the QF’s economy. Benefiting from the proximity to high-grade mineral deposits, these industries account for approximately 10% of the regional economy. Numerous industrial plants throughout the QF produce steel, pig iron, and metal alloys for domestic and international markets.

The intensive combination of mining, agriculture, and urban-industrial expansion has produced a highly fragmented and dynamic land use mosaic, exerting significant environmental pressure on local ecosystems and surface water quality throughout QF. In this context, existing environmental protection areas are essential for mitigating cumulative impacts on water quality, sediment dynamics, and the overall ecological integrity of the region’s watersheds.

## Materials and methods

### Sampling and laboratory analysis

The sampling network was designed based on 864 micro-basins (average area of 25 km^2^; 2nd–3rd order) delineated from ALOS/PALSAR digital elevation models. One sampling point was established near the outlet of each micro-basin. A total of 835 samples were collected during the rainy season (January–April 2023) and 845 during the dry season (August–October 2022), including 46 and 47 duplicate samples, respectively.

 The discrepancy in the number of sites is due to logistical constraints. All field and laboratory procedures were conducted by SGS Geosol LTDA (Vespasiano, MG) in accordance with the protocols of APHA (2017). Comprehensive details on sampling strategies, methodological structure, and quality control are provided in Medeiros Filho et al. (2025) and Almeida et al. (2025).

In the field, electrical conductivity, pH, dissolved oxygen, and temperature were measured using a multiparameter probe (HANNA HI98194 model), while turbidity was determined with a portable turbidimeter (HANNA HI98703 model).

In the laboratory, anions (Cl^−^, F^−^, NO_2_^−^, NO_3_^−^, H_2_S, and SO_4_^2−^) were quantified via ion chromatography, and total phosphorus was determined colorimetrically. Total, dissolved, and suspended solids were measured using standard gravimetric procedures. Metal concentrations were analyzed by inductively coupled plasma techniques: Al, Fe, and Mn were determined by optical emission spectrometry (ICP-OES), whereas a suite of trace elements (Ag, As, Ba, Be, B, Cd, Co, Cr, Cu, Hg, Li, Ni, Pb, Sb, Se, U, V, and Zn) was quantified by mass spectrometry (ICP-MS).

A rigorous quality assurance and quality control protocol, as detailed in Almeida et al. (2025), was applied throughout the analytical process to ensure data reliability and minimize analytical errors.

### Water quality index (WQI) calculation

Several methodologies have been developed for calculating Water Quality Indices (WQI), among which the approach proposed by the National Sanitation Foundation (NSF) in the United States of America during the 1970s is one of the most widely used. The NSF-WQI integrates key physical, chemical, and biological parameters, each assigned a specific weight (Appendix A) (Brown et al., 1970), providing a comprehensive assessment of water quality and supporting the interpretation of results as well as management and protection actions (Chidiac et al., 2023; Kachroud et al., 2019; Pereira, 2010; Uddin et al., 2018).

In Brazil, the NSF-WQI has been adapted by regional environmental agencies to better reflect local conditions. The São Paulo State Environmental Company (CETESB) modified the index in 1975 by prioritizing parameters related to domestic sewage contamination and public water supply, replacing nitrate and phosphate with total nitrogen and total phosphorus (CETESB, 2005). Similarly, the Minas Gerais Water Management Institute (IGAM) replaced total dissolved solids with total solids and adjusted parameter weights (IGAM, 2018). The main methodological differences between these indices are summarized in Appendix A.

The NSF-WQI, CETESB-WQI, and IGAM-WQI were calculated as the weighted product of normalized quality values for each parameter, according to Eq. 1.

1 $${\mathrm{WQI}} = \mathop \prod \limits_{i = 1}^{n} qi^{wi}$$

where: WQI = Water Quality Index, ranging from 0 to 100; q*i* = Normalized quality value for *i* parameter, derived from its respective quality curve; *wi* = Unitless weight assigned to the *i* parameter, reflecting its relative importance (0 ≤ *w* ≤ 1).

In 2001, the Canadian Council of Ministers of the Environment (CCME) introduced an alternative WQI methodology designed to standardize water quality classification under Canadian environmental conditions. A key distinction of the CCME-WQI is its flexibility, as it does not require a fixed set of parameters; instead, parameter selection is based on data availability and study-specific objectives (Dascalescu et al., 2017; Mostafaei, 2014; Salcedo-Sánchez et al., 2016). The index identifies parameters that exceed established thresholds and evaluates both the frequency and magnitude of these deviations. Additionally, it does not employ transformation functions or parameter weighting, characteristics that proponents argue make it a more universal and objective assessment tool (Salcedo-Sánchez et al., 2016).

The CCME-WQI is calculated by integrating three factors: Scope (F1), Frequency (F2), and Amplitude (F3), each ranging from 0 to 100. Scope (F1) represents the proportion of parameters that fail to meet water quality guidelines at least once (Eq. 2), while Frequency (F2) measures the proportion of individual tests that do not comply with these guidelines (Eq. 3). Amplitude (F3) quantifies the magnitude by which failed test values exceed the corresponding thresholds, normalized through an asymptotic function (Eq. 4). These components are combined to produce the final WQI (Eq. 5), where a scaling factor of 1.732 constrains results to a range from 0 to 100, with higher values indicating better water quality (Lumb et al., 2006).

2 $$F1 = \frac{n^\circ\,of\,non - compliant\,parameters}{{Total\,number\,of\,monitored\,parameters}} x 100$$

3 $$F2 = \frac{n^\circ\,of\,non - compliant\,analyses}{{total\,number\,of\,analyses performed}}x 100$$

4 $${\text{F3 }} = \frac{\sum n\Delta }{{0.01 x \sum n\Delta + 0.01 }} x 100$$

where $$\sum n\Delta = \frac{{\mathop \sum \nolimits_{i = 1}^{n} \left( {\frac{{\text{Non - compliant analysis value}}}{{\text{Standard value}}} - 1} \right)}}{{\text{total number of analyses performed}}}$$

5 $${\text{CCME - WQI}} = 100 {-} \left[ {\frac{{\sqrt {\left( {F1} \right)^{2} + \left( {F2} \right)^{2} + \left( {F3} \right)^{2} } }}{1,732}} \right]$$

This flexible and user-friendly structure makes the CCME-WQI a versatile tool for water quality assessment (Khan et al., 2005; Lumb et al., 2006). In this study, two CCME-WQI variants were calculated: (i) CCME1-WQI, based on the same parameter set used in the CETESB index; and (ii) CCME2-WQI, based on the inorganic parameters defined by CONAMA Resolution 357/2005, excluding cyanide and total residual chlorine, since they were not measured by the current research.

The Water Quality Index (WQI) results obtained from all methodologies were subsequently classified into five quality categories for interpretation and discussion: Excellent (EX), Good (GD), Regular (RG), Bad (BD), and Very Bad (VB). To improve readability and facilitate comparisons among the different indices, only the nomenclature of the quality classes was standardized and simplified, while the numerical classification intervals remained unchanged and consistent with those originally proposed by each methodology.

### Classification of microcatchment based on land use and land cover

**Fig. 2:**
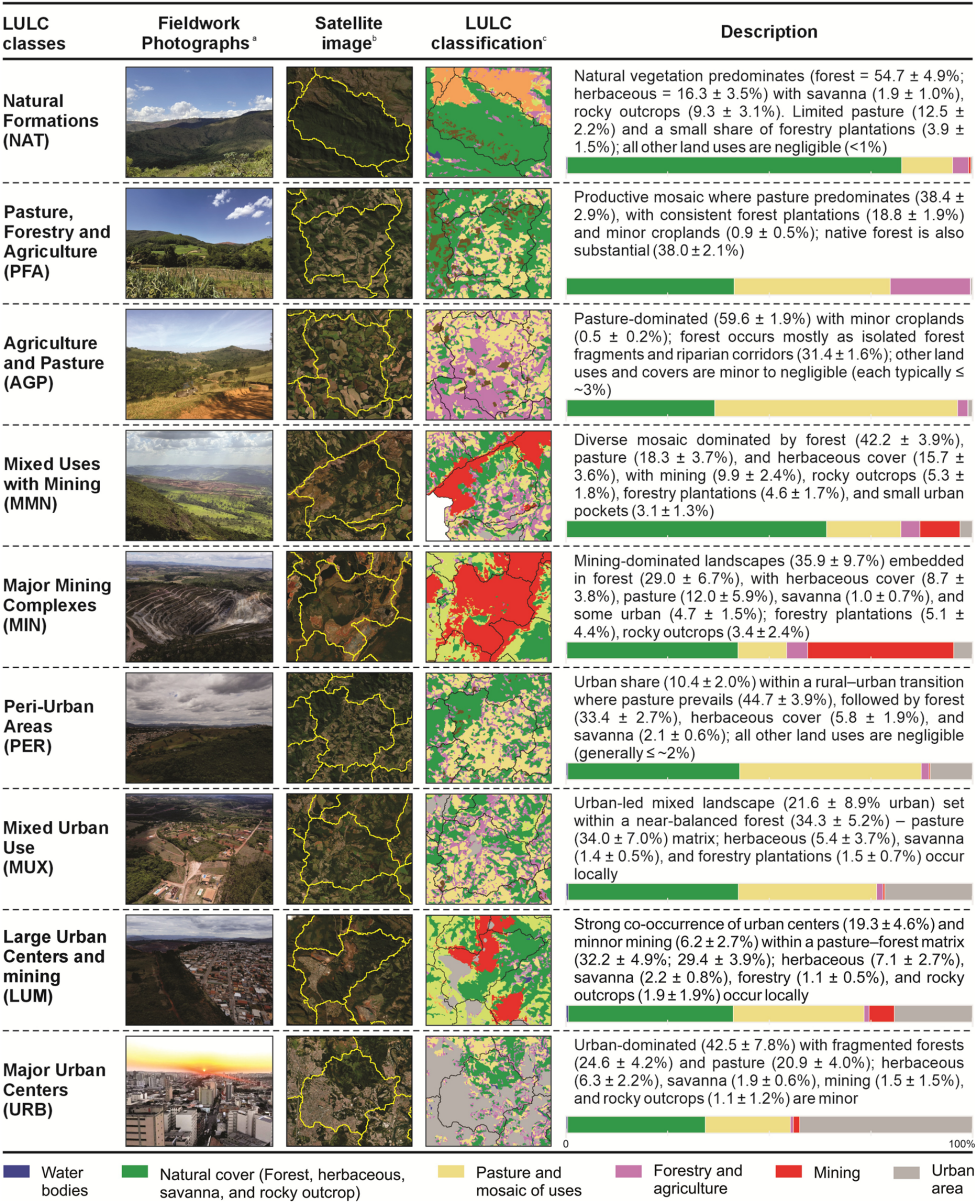
Summary of the land use and land cover (LULC) classification of microcatchments associated with surface water sampling sites. For each LULC class, the figure includes **a** field photograph characterizing the landscape, **b** a satellite image of a selected microcatchment, and **c** its corresponding LULC map. A brief description is presented for each case, along with a 100% stacked bar chart summarizing the mean proportional contribution of LULC types used for classification purposes. Source: **a** Fieldwork photographs taken by the authors (copyright-free). **b** Satellite imagery from Google Earth; map data © Google, Maxar Technologies (accessed on 10 September 2025). **c** LULC layer adapted from MapBiomas Project (2026)

The LULC classes were defined at the microcatchment scale using satellite imagery, the MapBiomas Project (2026) database, and field observations. A multi-step geoprocessing workflow (intersect and zonal statistics) was implemented in a geographic information system (GIS) to quantify the percentage of each LULC category within every microcatchment in the study area (Fig. 1). This procedure ensures an accurate representation of the landscape heterogeneity of the QF, where multiple land uses often coexist within a single spatial unit. Figure 2 illustrates the LULC classes recognized in the GIS environment and in fieldwork, with brief descriptions of their characteristics. Population data from the 2022 Demographic Census (IBGE, 2022) were integrated with the microcatchment boundaries to calculate population density for each drainage unit, enabling an evaluation of demographic pressure on water resources.

## Results and Discussion

### Seasonal and spatial variability of water quality

**Fig. 3:**
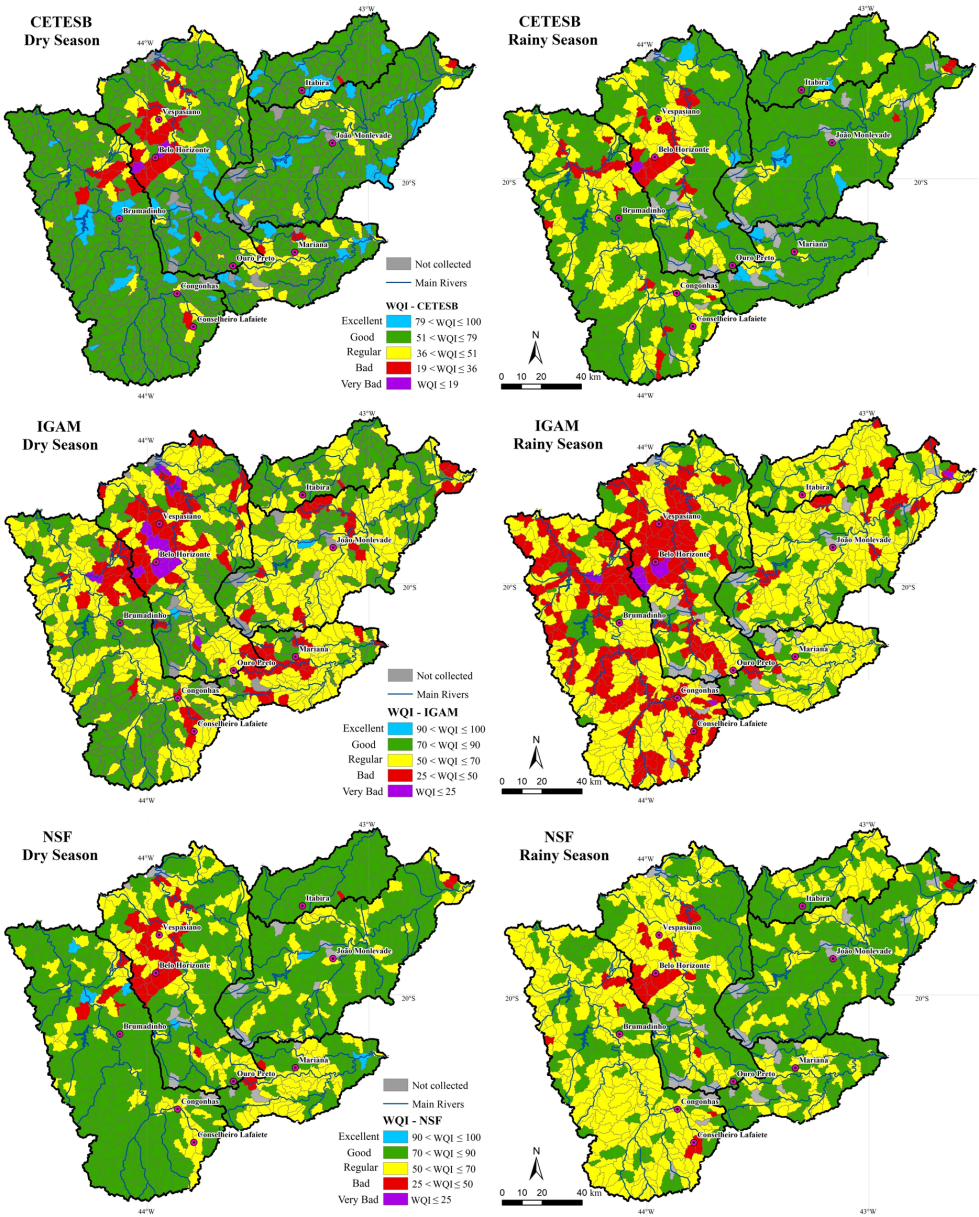
Spatial distribution of water quality indices (WQI) from São Paulo State Environmental Company (CETESB), Minas Gerais Water Management Institute (IGAM) and National Sanitation Foundation (NSF) methods, during the dry (left) and rainy (right) seasons

**Fig. 4:**
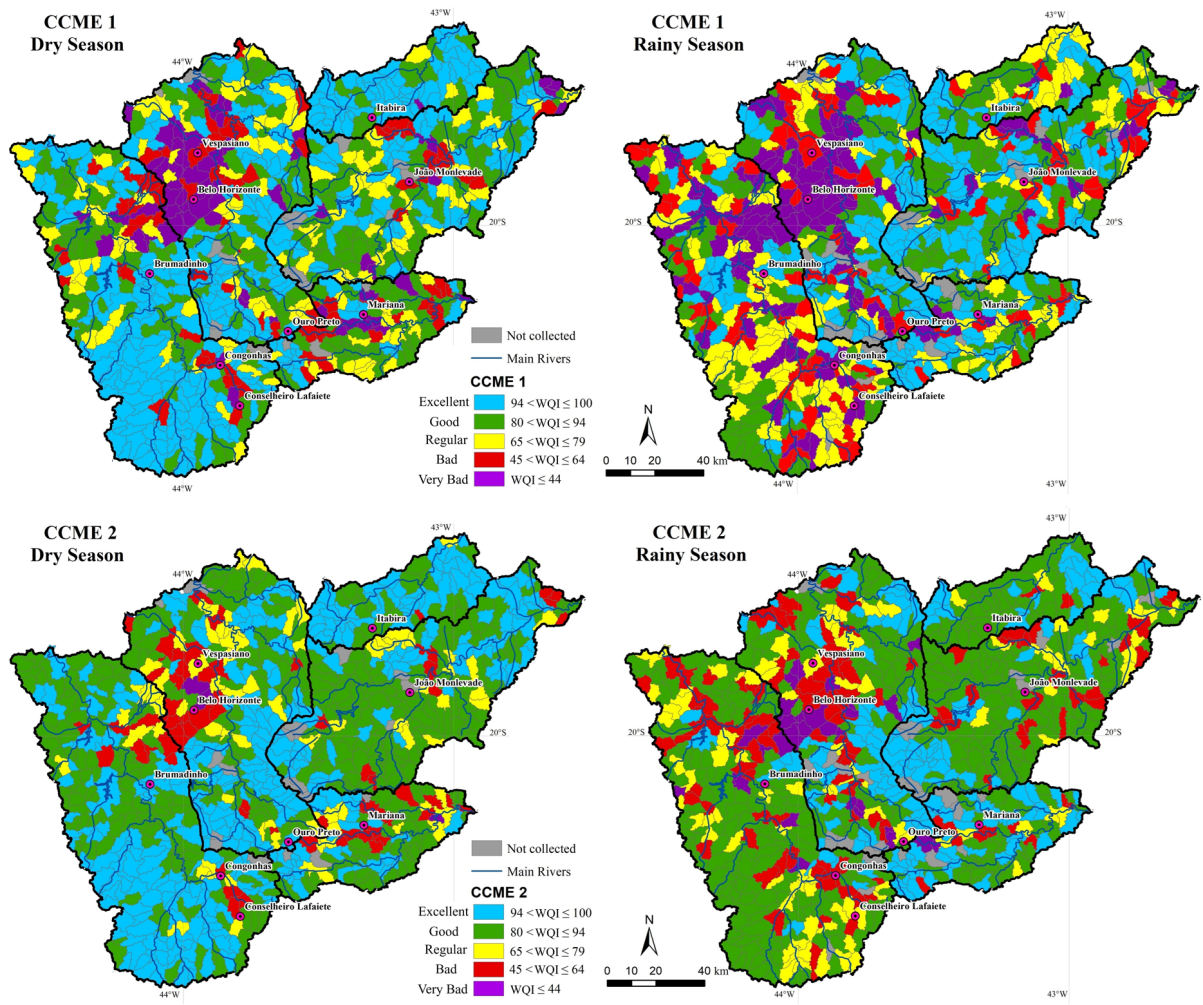
Spatial distribution of water quality indices (WQI) from CCME 1 and 2 methods, during the dry (left) and rainy (right) seasons

This study evaluated the results of five WQI approaches (CETESB, IGAM, NSF, and two CCME variants) to evaluate their effectiveness in capturing spatial and seasonal patterns of surface water quality in the QF. The spatial distribution of the indices is presented in Figs. 3 and 4, while Table 1 summarizes the statistical results for the dry (2022) and rainy (2023) seasons. Comparing different WQI methodologies is essential because each index employs distinct criteria and parameter weightings, which may lead to divergent water quality classifications (Lumb et al., 2011; Uddin et al., 2022). Such comparisons support the selection of context-appropriate assessment tools and facilitate the adaptation of WQI to local geographic, climatic, and socioeconomic conditions, resulting in more accurate and meaningful evaluations of water quality (Uddin et al., 2018).

**Table 1:** Descriptive statistics of water quality index (WQI) values and sample classifications counts for QF during the rainy (*n* = 835) and dry (*n* = 845) seasons

WQI Methods	Season	Descriptive Statistics					Number of samples within water quality classes				
		x̅	σ	M	Min	Max	Excellent	Good	Regular	Bad	Very Bad
CETESB	Rainy	59.5	12.0	62.0	18.0	90.0	11	609	182	32	1
	Dry	65.0	12.6	68.0	15.0	93.0	55	675	82	31	2
IGAM	Rainy	59.7	12.0	62.0	18.0	90.0	1	161	474	194	5
	Dry	65.9	12.7	69.0	15.0	95.0	5	352	388	92	8
NSF	Rainy	69.4	7.5	70.2	36.2	88.2	0	431	380	24	0
	Dry	72.7	9.4	74.5	26.6	93.6	6	596	212	31	0
CCME1^a^	Rainy	76.1	22.6	84.2	16.3	100.0	217	221	153	116	128
	Dry	83.9	19.6	89.78	19.0	100.0	337	240	126	76	66
CCME2^b^	Rainy	82.7	16.1	89.1	39.6	100.0	167	423	92	123	30
	Dry	89.2	12.1	92.8	39.3	100.0	329	377	73	63	3

x̅ = Mean; σ = standard deviation; M = Median; Min = Minimum; Max = Maximum; ‘^a^’ Considering the same parameters from CETESB (2005); ‘^b^’ Considering selected parameters contemplated in CONAMA (2005). CETESB—São Paulo State Environmental Company, IGAM—Minas Gerais Water Management Institute, NSF—National Sanitation Foundation, CCME – Canadian Council of Ministers of the Environment

The results indicate substantial differences among the indices, highlighting their varying sensitivity. The CETESB-WQI yielded the highest frequency of GD classifications (Table 1), with the proportion of samples increasing from 72.9% in the rainy season to 79.9% in the dry season. The proportion of RG samples decreased from 21.8% to 9.7%, while BD and VB classifications remained low (BD ≈ 4%; VB = 0.1% rainy, 0.2% dry), occurring primarily in spatially restricted areas (Fig. 3).

In contrast, the IGAM-WQI provided a more conservative evaluation, indicating greater water quality impairment (Table 1). During the rainy season, most samples (56.7%) were classified as RG, with a significant portion (23.2%) as BD, and only 19.3% as GD. This aligns with broader concerns about the environmental consequences of economic development on water resources (Javed et al., 2017; Marques et al., 2007). Water quality improved during the dry season, with the GD category rising to 41.7% and the BD category falling to 10.9%. The VB classification was present in both seasons (0.6% rainy, 1% dry). The broader spatial extent of degraded water quality according to the IGAM index is apparent in Fig. 3. These findings underscore that WQI, while a simplified method to express water quality for various uses, have inherent limitations that can lead to divergent interpretations of the same environment (Lumb et al., 2011; Uddin et al., 2018).

The NSF-WQI results indicate moderate water quality, with higher WQI values during the dry season. In the rainy season, samples were predominantly classified as GD (51.6%) and RG (45.5%). In the dry season, a marked shift occurred, with the proportion of GD samples increasing to 70.5% and RG decreasing to 25.1%. The consistent absence of EX and VB classifications in both seasons suggests that the index portrays a system without extreme water conditions, but with noticeable seasonal variability.

In contrast, the flexible CCME-WQI resulted in a higher proportion of samples classified in upper quality categories, particularly when using parameters from CONAMA 357. Both CCME variants showed a strong seasonal improvement, with a significant reduction in BD and VB classifications during the dry season. The CCME1-WQI (CETESB parameters) classified a notable portion of samples as EX (26% rainy, 39.9% dry), while the CCME2-WQI (CONAMA parameters) was even more optimistic, showing the highest overall quality with most samples in the GD category (50.7% rainy, 44.6% dry) and minimal VB (3.6% rainy, 0.4% dry) classifications.

Spatially, all methodologies consistently identified the metropolitan region of Belo Horizonte as the area with the most critical water quality conditions (Figs. 3 and 4). As the third-largest urban agglomeration in Brazil (IBGE, 2019) and a major global conurbation (UN, 2018), this region exemplifies the complex interplay of socioeconomic and environmental pressures that contribute to the degradation of its primary rivers, the Velhas and the Paraopeba. These pressures, driven by intense urbanization, industrial and mining activities, and persistent gaps in sanitation infrastructure, help explain the spatial concentration of poor water quality observed across all indices.

Microcatchments in the southwestern portion of the QF, particularly in the municipalities of Conselheiro Lafaiete and Congonhas, showed persistently poor water quality across all methodologies. The Maranhão River, one of the main tributaries of the Paraopeba, stands out as a documented pollution hotspot, reflecting the cumulative effects of mining operations, unplanned urban expansion, and severe deficits in sanitation infrastructure. These pressures are further intensified by the degradation of riparian vegetation and irregular riverbank occupation, which contribute to habitat loss and declining water quality.

In contrast, the eastern portion of the QF generally exhibited more favorable water quality across all methodologies (Figs. 3 and 4). Exceptions occurred in watersheds influenced by major industrial and urban centers, including the municipalities of João Monlevade, Itabira, and Ipatinga, where the impacted waters of the Piracicaba River converge with the Doce River.

**Fig. 5:**
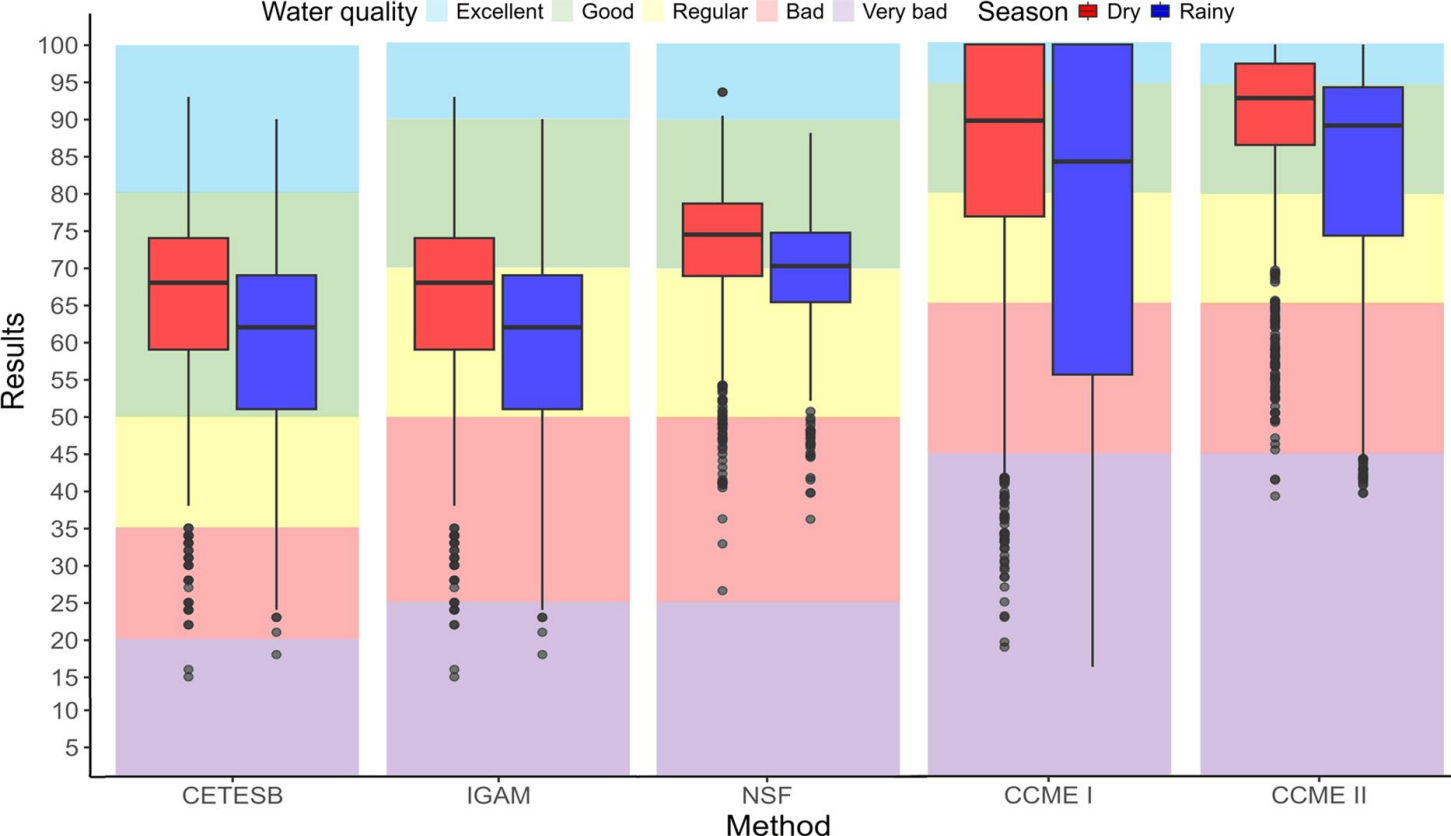
Boxplot comparison of water quality index (WQI) results during dry (red) and rainy (blue) seasons calculated using different methods (CETESB, IGAM, NSF, and CCME). Detailed information about each method is provided in topic 3.2

The seasonal analysis summarized in Fig. 5 reveals an increase in water quality during the dry season. The CETESB-WQI illustrates this trend well: during the rainy season, most watersheds fall within the GD class, with BD conditions concentrated around the Belo Horizonte metropolitan area. In the dry season, water quality improves markedly, with a substantial increase in watersheds classified as EX. This pattern, also evident in the spatial distributions shown in Figs. 3 and 4, reflects the combined effects of seasonal dilution and pollutant wash-off during the rainy period.

A comparative evaluation of the methodologies reveals distinct classification behaviors and sensitivities. CETESB-WQI is the least stringent, particularly for BD and VB waters, a pattern that likely reflects its design for Brazilian conditions, where widespread domestic sewage and industrial discharge are common. IGAM-WQI and NSF-WQI yielded more conservative assessments, with large areas classified as RG or BD during the rainy season. Their broader intervals for lower-quality classes make them more sensitive to anthropogenic pressures.

The CCME-WQI, in contrast, produced more optimistic classifications. Because the method evaluates only parameters that exceed legal thresholds and excludes compliant parameters from the calculation, its aggregation procedure tends to smooth the influence of isolated high values. CCME1-WQI (CETESB parameters) and CCME2-WQI (CONAMA parameters) followed the expected seasonal pattern, indicating lower quality during the rainy season. Although the CCME2-WQI incorporates metals regulated by CONAMA and the QF region exhibits natural geochemical enrichment in several of these elements, this enrichment was not captured by the resulting water quality classifications.

The comparison of water quality indices indicates that their applicability is strongly dependent on the study objectives and the environmental context under evaluation. The CETESB-WQI, IGAM-WQI, and NSF-WQI are characterized by methodological simplicity and high sensitivity to organic pollution and eutrophication, making them effective in areas influenced by domestic wastewater, although they show limited capacity to assess inorganic contaminants. In contrast, the CCME-WQI offers greater methodological flexibility by incorporating multiple parameters and regulatory thresholds, rendering it more suitable for complex environments affected by both natural and anthropogenic contamination sources. However, its structure, which is based on the number and magnitude of guideline exceedances, tends to yield more optimistic classifications when exceedances are sporadic. Consequently, high CCME-WQI values should not be interpreted as evidence of low environmental impact but rather as a reflection of a compliance-oriented methodological framework, whereas the other indices classify continuous gradients of water quality degradation more conservatively.

### Analysis of LULC catchment classification and its application to water quality

The LULC are distinct yet related concepts. Land use refers to the functional ways humans utilize the landscape (e.g., agriculture, mining, urban development), whereas land cover describes the physical characteristics of the Earth’s surface (e.g., forests, water bodies, built-up areas) (Heidkamp & Christian, 2022). Anthropogenic LULC changes, such as deforestation and urban expansion, generate substantial environmental impacts, including biodiversity loss, water quality degradation, and alterations to hydrological regimes (Henderson & Christian, 2022; Taylor & Rising, 2021). Integrating WQI with LULC analysis is therefore essential for diagnosing the condition of aquatic ecosystems and linking water quality patterns to specific human-induced pressures (Pandey et al., 2023).

**Fig. 6:**
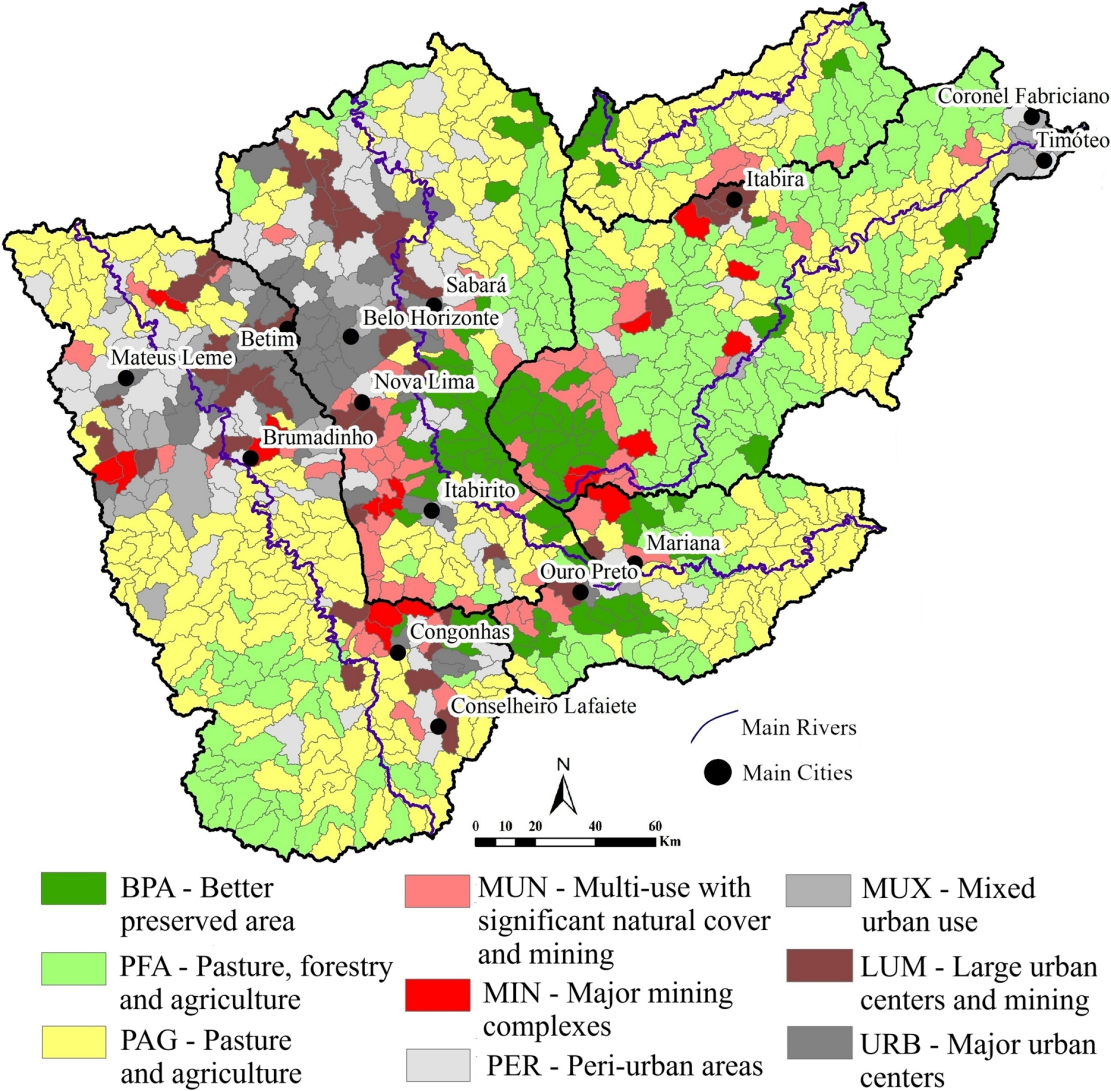
Spatial distribution of land use and land cover (LULC) classes across microcatchments in the Iron Quadrangle (QF), southeastern Brazil. Each microcatchment was classified according to the LULC composition defined in this study, based on a reclassification of the MapBiomas dataset. See Fig. 6 for visual examples and LULC class descriptions

Nine LULC classes were identified in the study area (Fig. 2): Better preserved areas (BPA); Pasture, forestry and agriculture (PFA); Pasture and agriculture (PAG); Multi-use with significant natural cover and mining (MUN), Major mining complexes (MIN); Peri-urban areas (PER), Mixed urban use (MUX), Large urban centers and mining (LUM), and Major urban centers (URB). The LULC classification for the QF’s microcatchments revealed a landscape of striking contrasts, distinguishing densely urbanized zones, large-scale mining areas, agricultural mosaics, and remnants of natural vegetation. Figure 6 illustrates the spatial distribution of the defined classes.

The proposed LULC classification reveals a complex and heterogeneous landscape mosaic across the QF (Fig. 6). BPA represents areas with minimal anthropogenic interference, exhibiting high proportions of natural cover (≈ 82%), including forests, savannas, grasslands, and rock outcrops. These BPA areas form ecological corridors and preservation zones concentrated in the central QF, typically associated with quartzitic and itabiritic ridge systems and headwater zones, which naturally restrict the expansion of anthropogenic activities. In contrast, two predominant anthropogenic mosaics stand out: PAG, dominated by agricultural and livestock activities (≈ 60% coverage), and PFA, where silviculture frequently coexists with agriculture and pasture (≈ 58%). The PAG and PFA classes function as transitional areas between these natural remnants and the core urban–mining centers located mainly in the southwestern and eastern sectors of the QF.

Among the LULC classes mainly influenced by anthropogenic activities, URB exhibit high levels of urbanization (average 42%), while MIN are characterized by extensive mining operations (average 37%). Where mining districts and urban areas intersect, the LUM class was identified, reflecting integrated patterns of industrial activity and urban development (averaging 16% urbanization and 6% mining).

Areas under more diffuse anthropogenic influence, particularly from human occupation, were observed into three classes (Fig. 6): i) MUN, which comprises areas where multiple anthropogenic uses (including minor mining activity) co-occur with natural covers, typically dominated by natural formations (≈ 64%); ii) MUX represents landscapes where urbanization (≈ 21%) is associated with non-mining uses, particularly natural cover (≈ 42%) and agricultural–pasture mosaics; iii) PER delineates rural–urban transition zones characterized by dispersed settlements and a strong presence of agricultural and pasture lands (≈ 47%). This detailed LULC classification provides the foundational framework for analyzing its direct relationship with water quality, as explored in the following section.

The main regions of mining operations are clearly delineated. The MIN and MUN classes are concentrated in the principal mining hubs of the QF, including the municipalities of Itabira, Ouro Preto, Mariana, and Brumadinho (Fig. 6). The MUN class typically surrounds the MIN zones, forming transitional regions that integrate pastures, urban areas, and mining infrastructure. The primary urban–industrial regions occur in the northwest, within the Belo Horizonte Metropolitan Region, and in the eastern Vale do Aço, in municipalities such as Coronel Fabriciano and Timóteo (Fig. 6). These areas are dominated by the URB and LUM classes, reflecting economies strongly linked to steel and metallurgical production. At the margins of these urban cores, the PER and MUX classes form a transitional fringe characterized by low-density urban expansion, small-scale mining, and rural land uses.

**Fig. 7:**
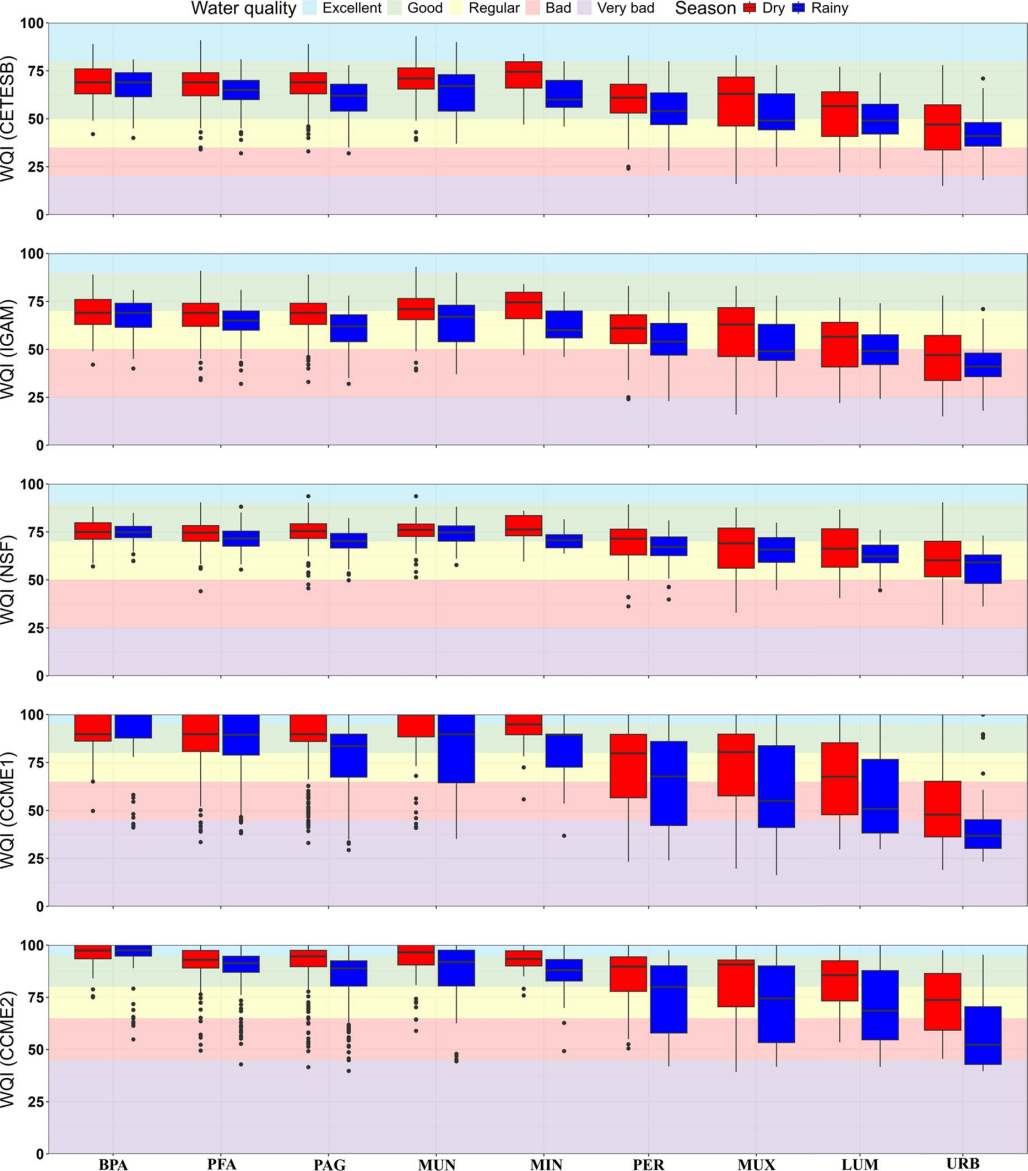
Boxplot comparison of Water Quality Index (WQI) results from five methodologies across different land use and land cover (LULC) classes during dry and rainy seasons. See Fig. 2 for visual examples and LULC class descriptions, and Fig. 6 for the spatial distribution of each LULC class

Figure 7 compares WQI values across LULC classes described above during dry and rainy seasons, integrating results from all five WQI methods. A detailed descriptive statistic for each class is provided in Appendix B to support a more detailed inspection. Overall, the boxplots reveal a clear and consistent gradient of water-quality deterioration with increasing anthropogenic intensity, largely independent of the WQI method applied. Classes dominated by natural cover and low-intensity rural land use (BPA, PFA, and PAG) consistently showed the highest WQI values; notably, MUN and MIN were also toward the higher-quality end of the distribution. In contrast, the most human-impacted settings (URB, LUM, MUX, and PER) exhibited the lowest scores. This pattern is even more predominant when comparing WQI distribution maps (Figs. 3 and 4) and LULC classification (Fig. 6) proposed herein.

This left-to-right decline in WQI (Fig. 7) mirrors the shift from predominantly natural landscapes to areas increasingly influenced by major urban centers, industrial activities, and other intensive land uses, indicating that degradation is primarily driven by pollution sources associated with human-dominated environments.

To statistically verify whether the observed differences among classes were significant, Dunn’s test was applied (Appendix C) to conduct pairwise comparisons between LULC classes for each WQI method and season. Across most WQI methodologies during the dry season, BPA, PFA, PAG, MUN, and MIN did not differ significantly from one another (*p* ≥ 0.05), reinforcing that these classes cluster at the higher-quality end of the gradient. During the rainy season, this pattern partly shifts, most notably for PAG, indicating that seasonal runoff can alter class separation even within relatively low-intensity settings. In contrast, PER, MUX, LUM, and URB were consistently and significantly different (*p* < 0.05) from the above-mentioned group in both seasons, supporting the interpretation of poorer water quality under more human-impacted land uses. Nevertheless, pairwise comparisons also suggest that, in some cases, WQI values can be statistically equivalent among PER, MUX, LUM, and URB, reflecting overlapping levels of impact across these four anthropogenic classes depending on the method and season.

Despite this general trend, relevant methodological differences were observed among the indices. The CETESB-WQI, IGAM-WQI, and NSF-WQI were more sensitive to gradual variations in land-use composition, conservatively discriminating intermediate classes and capturing increases in dispersion (cf. deviation metrics in Appendix B), particularly during the rainy season, when pollutant mobilization and transport are intensified. In contrast, both CCME-WQI formulations yielded systematically higher median values across nearly all classes, indicating lower sensitivity to anthropogenic impacts when exceedances of regulatory thresholds are sporadic.

In addition to methodological contrasts, the seasonal analysis demonstrates that differences between rainy and dry periods are most pronounced in land-use classes with higher proportions of impervious surfaces and reduced vegetation cover, highlighting the role of hydrological processes in controlling water quality. All WQI methods consistently identified intensified anthropogenic impacts during the rainy season, characterized by reduced WQI values, especially in urban and mixed urban classes (URB, LUM, MUX, and PER). Conversely, classes with greater natural cover (BPA, PFA, and PAG) exhibited lower seasonal variability (Fig. 7), reflecting enhanced retention capacity and hydrological buffering. During the dry season, WQI values were generally higher and less dispersed across most LULC classes, indicating reduced surface runoff and diminished mobilization of diffuse pollutants; nevertheless, highly anthropogenic areas maintained lower WQI values than more preserved classes, demonstrating that anthropogenic pressures persist even in the absence of precipitation. These findings reinforce that the dry season represents a more stable hydrological condition, under which the structural effects of land use on surface water quality become more evident.

BPA consistently exhibited the highest medians and lowest dispersion, with values concentrated in the EX–GD range. These areas, comprising forests, savannas, grasslands, and rock outcrops, function as hydrogeochemical benchmarks by regulating runoff, reducing erosion, filtering pollutants, and maintaining stable water chemistry (Sun et al., 2020; Cheng et al., 2022; Caldwell et al., 2023). However, grouping all-natural vegetation into a single class introduces limitations, since native forests retain contaminants more effectively than pasture-dominated natural covers (Chen et al., 2021; Zhou et al., 2022). Ongoing fragmentation of these vegetated areas further diminishes their capacity to protect water resources from diffuse pollution (Mello et al., 2022; Zhang et al., 2021). Therefore, maintaining sufficient and connected forested areas remains essential for safeguarding watershed integrity and water quality (Caldwell et al., 2023; Piffer et al., 2021).

As natural vegetation becomes progressively replaced by agroforestry uses, a mild to moderate decline in water quality emerges. The PFA and PAG classes show WQI medians within the GD to RG range, with degradation becoming more pronounced during the rainy season due to diffuse pressures such as soil disturbance, fertilization, and cattle trampling. These processes enhance the transport of turbidity, total solids, nutrients, and coliforms to rivers (Toledo & Nicolella, 2002). Agricultural inputs promote eutrophication and pesticide contamination, while pastures contribute to soil compaction and microbiological loading. Depending on management practices, forestry activities can modify hydrological regimes and increase erosion or nutrient exports (Dallas & Day, 2004; Duffy et al., 2020). Together, these pressures explain the moderate yet consistent reduction in water quality relative to natural areas.

The MUN and MIN, although both influenced by mining activities, exhibit distinct water quality behaviors primarily controlled by differences in land-cover composition. In MUN, the high proportion of natural vegetation (cf. Figure 2) acts as an effective buffering factor, resulting in the highest median WQI values across nearly all methodologies and both hydrological periods (Fig. 7). The CETESB, IGAM, and NSF indices consistently classify these waters as GD, with a slight improvement during the dry season, while the CCME-WQI, particularly the CCME-2 variant, indicates GD to EX conditions, reflecting high compliance with water-quality standards (Fig. 7). In contrast, MIN shows a moderate decline in WQI values, especially during the rainy season, when intensified surface runoff increases the transport of suspended sediments, fine particulates, and mine-related contact waters to the drainage network. This seasonal pulse tends to elevate turbidity and total solids and can enhance the mobilization of particle-bound and dissolved PTEs (Peters et al., 2005; Chapman et al., 2020), thereby reducing index scores across methods. During the dry period, reduced diffuse inputs lead to a relative recovery in water quality, particularly as captured by the CCME-based indices.

Classes associated with diffuse urbanization (PER and MUX) exhibit intermediate water-quality conditions but with high variability, reflecting the combined influence of agricultural, natural, and urban land uses. In PER, the dominance of agricultural and pasture lands coupled with dispersed urban occupation imposes strong seasonal control, with marked degradation during the rainy season due to runoff-driven transport of nutrients, organic matter, and sediments. The MUX represents a heterogeneous land-use mosaic with moderate urbanization and mixed natural and agro-pastoral cover, explaining its intermediate WQI values and the instability observed across methodologies and seasons. In both classes, multiple diffuse sources act simultaneously, hindering the identification of a single dominant controlling factor and reinforcing hydrochemical heterogeneity.

The most critical conditions are observed in LUM and URB, where urban pressures dominate water-quality degradation. In LUM, where mining districts spatially overlap with urban areas, low median WQI values and high dispersion indicate severe and spatially heterogeneous degradation driven primarily by untreated or partially treated urban effluents and intensified runoff over impervious surfaces. These processes disproportionately affect parameters related to nutrients, organic matter, microbial contamination, and dissolved oxygen, which are heavily weighted in the CETESB, IGAM, and NSF indices, explaining their predominant classification of waters as RG to BD. The influence of mining becomes evident mainly in the CCME-WQI when metallic parameters are explicitly incorporated (CCME2), highlighting its more specific and parameter-dependent role. In URB, this pattern intensifies, with continuous pollutant inputs and reduced dissolved oxygen leading to BD to VB classifications across all methodologies and seasons.

Overall, the water-quality gradient across LULC classes can be summarized as:

BFA > (PFA ≳ PAG) ≳ MUN > MIN ≳ (PER ≳ MUX) > LUM > URB, confirming that the preservation of natural land cover plays a fundamental role in maintaining surface-water quality, even in mining-influenced settings. These findings emphasize the need for differentiated management strategies, including the protection of remaining natural areas, enhanced sediment and metal control in mining districts, conservation practices in peri-urban and agropastoral zones, and the prioritization of sanitation and stormwater infrastructure in urban environments.

### Integrated analysis of population distribution and water quality

Population density is a key driver of water quality degradation, particularly in urbanized watersheds (Tromboni & Dodds, 2017; Tromboni et al., 2021). In the QF, this relationship is evident: microcatchments with a higher proportion of urban area, those classified as URB, LUM, MUX, and PER (Fig. 6), consistently exhibit poorer WQI results than less populated areas (Fig. 7). Elevated population concentrations intensify impervious surface cover, untreated sewage discharge, and diffuse nutrient and organic-matter inputs, all of which degrade physicochemical and biological water parameters (Chen et al., 2016; Rimba et al., 2021).

**Fig. 8:**
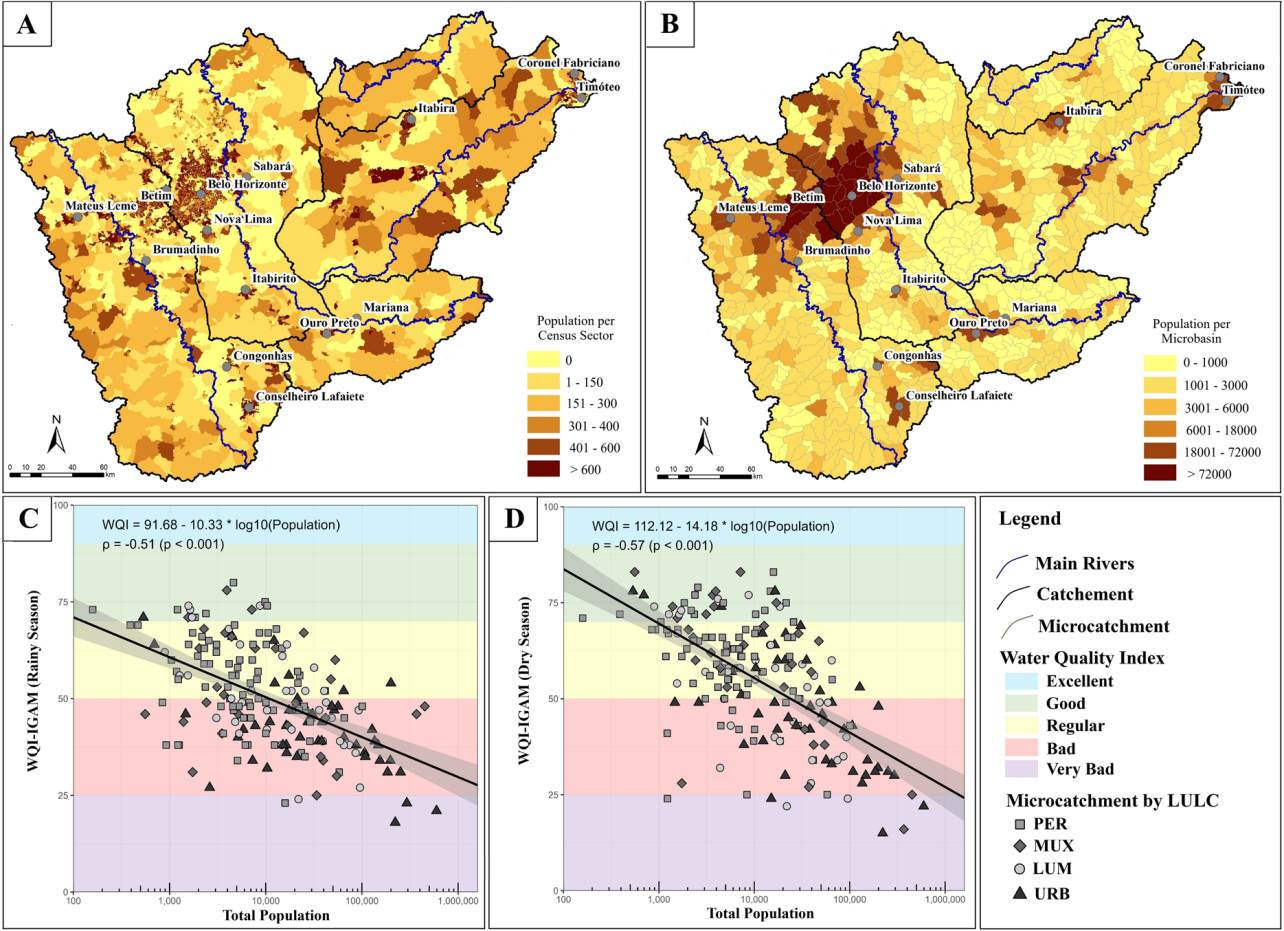
Relationship between population distribution and surface-water quality in the Quadrilátero Ferrífero. **A** Population density map by census sector; **B** Total population aggregated within each micro-catchment; **C**–**D** Relationship between log_10_-transformed total population and IGAM-WQI using Spearman’s rank correlation during the **C** rainy season (*ρ* = − 0.51, *p* < 0.001) and **D** dry season (*ρ* = − 0.57, *p* < 0.001); solid lines show linear regression fits for visualization

Figure 8A highlights dense urban nuclei within the Belo Horizonte Metropolitan Region (e.g., Betim, Contagem, Belo Horizonte) and other major mining-industrial centers (e.g., Congonhas, Itabira, João Monlevade). When aggregated by micro-basin (Fig. 8B), these data clearly identify critical zones where populations exceeding 18,000 inhabitants coincide with intense land-use pressures, indicating a high potential for water quality impairment.

A robust, monotonic negative correlation exists between total population per micro-basin and IGAM-WQI (rainy season: *ρ* = –0.51; dry season: *ρ* = –0.57; *p* < 0.001), confirming that demographic pressure systematically drives water quality degradation (Fig. 8C and D). The slightly stronger relationship in the dry season reflects lower dilution capacity and the dominance of continuous point-source inputs, whereas in the rainy season the “first flush” effect mobilizes pulses of contaminants, including fines, adsorbed metals, nutrients, and fecal coliforms, from impervious surfaces, strongly depressing IGAM-WQI due to its sensitivity to turbidity and coliform indicators.

Stratifying microcatchments by LULC refines this diagnosis. PER and MUX exhibit greater dispersion, with some micro-basins maintaining GD quality where riparian vegetation remains extensive. In contrast, LUM and URB cluster consistently within the RG to VB range, reflecting the combined pressures of high population density, imperviousness, deficient sanitation, and intense hydrologic connectivity to pollutant sources. Practically, the probability of poor water quality (WQI ≤ 45) rises sharply above ~ 10,000 inhabitants and becomes nearly certain above ~ 100,000.

Together, these patterns reveal a clear demographic gradient of water quality deterioration across QF. Although the seasonal mechanisms differ, runoff-driven degradation in the wet season versus concentration-driven impacts in the dry, the outcome is the same: increasing population density predictably reduces WQI. These findings underscore the urgent need for integrated territorial planning, improved drainage and sanitation infrastructure, and the restoration of riparian and natural areas to safeguard water resources in the QF.

## Conclusion

This study demonstrates that surface water quality in the QF is strongly associated with LULC, following a consistent gradient from predominantly natural environments to areas characterized by greater urban influence. Although all four WQI methodologies applied in this study captured this general pattern, their classifications differed according to their parameter sensitivity and conceptual conceptual structure. The CETESB, IGAM, and NSF indices appeared more responsive to eutrophication and organic pollution whereas their ability to reflect PTE-related impacts was comparatively lower. In contrast, the CCME-WQI, particularly the variant incorporating PTE variables, showed greater potential for application in mining-influenced regions due to its flexibility in incorporating inorganic pollutants.

Spatial analyses revealed that microcatchments dominated by BPA generally exhibited the highest water quality values, whereas lower classifications were more frequently associated with microcatchments influenced by urban land uses. Additionally, the observed negative correlation between total population and water quality (*ρ *≈ −0.55) conditions, potentially reflecting the combined influence of urbanization, sanitation deficits, and other anthropogenic pressures. Seasonal analyses also indicated generally higher water quality values during the dry season, whereas the rainy season was associated with greater variability, likely reflecting the influence of hydrological processes such as runoff, dilution, and diffuse pollutant transport. These findings reinforce the importance of considering both land-use patterns and seasonal dynamics in water quality assessments and monitoring programs.

From a management perspective, the results suggest that the conservation of native vegetation, particularly in headwater areas and riparian zones, may contribute to the maintenance of favorable water quality conditions. Likewise, improvements in sanitation infrastructure, urban drainage systems, and land-use planning represent important measures for reducing anthropogenic pressures on aquatic systems. Among the evaluated methodologies, the CCME-WQI may provide a useful framework for regional monitoring programs in mining-dominated areas, especially when combined with complementary indices such as CETESB or IGAM that are more sensitive to eutrophication and sediment-related impacts.

Overall, the results highlight the importance of integrated monitoring and management strategies capable of addressing the combined influences of land use, population density, seasonal hydrological variability, and mining activities on surface water quality in the QF.

## Supplementary Information

Below is the link to the electronic supplementary material.

Supplementary file1 (DOCX 247 KB)

## Data Availability

The surface water data that support the findings of this study were collected and analyzed under the framework of a research project conducted in partnership with Instituto Tecnológico Vale (ITV), Belém, Brazil. The data are not publicly available as they were generated under institutional agreements and remain under the stewardship of ITV. The data are, however, available upon reasonable request and with the permission of Instituto Tecnológico Vale (ITV).
