# Supplementary material for: Land use controls the spatiotemporal patterns of surface water quality of the Quadrilátero Ferrífero mineral province, Brazil
Source: Environ Geochem Health. 2026 Jul 20;48(11):474. doi: 10.1007/s10653-026-03358-7 (PMC13385133; doi:10.1007/s10653-026-03358-7)
Supplement: Supplementary file 1 — Supplementary file1 (DOCX 247 KB) [file 10653_2026_3358_MOESM1_ESM.docx]

**Appendices**

**Manuscript Title:** Land use controls spatiotemporal patterns of surface water quality in a world-class Brazilian mineral province

**Authors:** Gabriel Soares de Almeida*, Rafael Tarantino Amarante, Normara Yane Mar da Costa, Roberto Dall’Agnol, Prafulla Kumar Sahoo, Paulo Rógenes Monteiro Pontes, Emmanoel Vieira Silva Filho, Eduardo Duarte Marques, Raquel Fernandes Mendonça, Abraão Gomes Soares Junior, Gabriel Negreiros Salomão

***Corresponding author email:** gabriel.almeida@pq.itv.org

[**Appendix A -** Parameters and their assigned weights in the NSF, CETESB, and IGAM Water Quality Index (WQI) methodologies. 2](#_Toc222490170)

[**Appendix B** - Descriptive statistics of Water Quality Index (WQI) values for land use and land cover (LULC) classes in the Quadrilátero Ferrífero region, calculated using various methods during dry and rainy seasons. 3](#_Toc222490171)

[**Appendix C** - Results of Dunn’s test for pairwise comparisons among land use and land cover (LULC) classes, considering different water quality index (WQI) methodologies. and seasonal conditions (dry and rainy periods). Red cells indicate statistically significant differences (ρ < 0.05), while blue cells indicate no significant differences (ρ ≥ 0.05). LULC classes: BPA = Better preserved areas; PFA = Pasture, forestry and agriculture; PAG = Pasture and agriculture; MUN = Multi-use with significant natural cover and mining; MIN = Major mining complexes; PER = Peri-urban areas; MUX = Mixed urban use; LUM = Large urban centers and mining; URB = Major urban centers. 5](#_Toc222490172)

# **Appendix A -** Parameters and their assigned weights in the NSF, CETESB, and IGAM Water Quality Index (WQI) methodologies.

| **Parâmetros** | **Unit** | **Weights (w)** | | |
| --- | --- | --- | --- | --- |
|  |  | **CETESB** | **IGAM** | **NSF** |
| Dissolved Oxygen | mg/L | 0.17 | 0.17 | 0.17 |
| Thermotolerant Coliforms | NMP/100mL | 0.15 | 0.15 | 0.16 |
| pH | - | 0.12 | 0.12 | 0.11 |
| Biochemical Oxygen Demand | mg/L | 0.10 | 0.10 | 0.11 |
| Water Temperature | ºC | 0.10 | 0.10 | 0.10 |
| Total Nitrogen | mg/L | 0.10 | - | - |
| Nitrate | mg/L | - | 0.10 | 0.10 |
| Total Phosphate | mg/L | 0.10 | 0.10 | 0.10 |
| Turbidity | NTU | 0.08 | 0.08 | 0.08 |
| Total Dissolved Solids | mg/L | - | - | 0.07 |
| Total Solids | mg/L | 0.08 | 0.08 | - |

**CETESB** – Companhia de Tecnologia de Saneamento Ambiental, 2005. Relatório de águas interiores do Estado de São Paulo de 2005. Available in: <http://www.cetesb. sp.gov.br/Agua> Accessed on 02 jan. 2025.

**IGAM** - Instituto Mineiro de Gestão das Águas, 2018. Índice de Qualidade Das Águas – IQA. Available in: <https://igam.mg.gov.br/w/indice-de-qualidade-das-aguas-iqa>. Accessed on 04 jan 2025.

**NSF** - Brown, R. M., McClelland, N. I., Deininger, R. A., & Tozer, R. G. (1970). A water quality index-do we dare. Water and sewage works, 117(10).

# **Appendix B** - Descriptive statistics of Water Quality Index (WQI) values for land use and land cover (LULC) classes in the Quadrilátero Ferrífero region, calculated using various methods during dry and rainy seasons.

| **LULC**  **Classes^a^** | **WQI Methods^b^** | **Season** | **n** | **x̅** | **σ** | **CV** | **MAD** | **Min** | **P25** | **P50** | **P75** | **P90** | **P95** | **P98** | **Max** | **Skew** | **Rku** | **pSW** |
| --- | --- | --- | --- | --- | --- | --- | --- | --- | --- | --- | --- | --- | --- | --- | --- | --- | --- | --- |
| **BPA** | CETESB | Dry | 67 | 66.5 | 10.5 | 0.16 | 8.9 | 40.0 | 61.5 | 69.0 | 74.0 | 78.0 | 78.7 | 80.0 | 81.0 | -0.85 | 2.65 | <0.05 |
|  |  | Rainy | 70 | 69.2 | 9.8 | 0.14 | 10.4 | 42.0 | 63.0 | 69.0 | 76.0 | 81.0 | 82.6 | 86.5 | 89.0 | -0.36 | 2.84 | 0.48 |
|  | IGAM | Dry | 67 | 66.5 | 10.5 | 0.16 | 8.9 | 40.0 | 61.5 | 69.0 | 74.0 | 78.0 | 78.7 | 80.0 | 81.0 | -0.85 | 2.65 | <0.05 |
|  |  | Rainy | 70 | 69.2 | 9.8 | 0.14 | 10.4 | 42.0 | 63.0 | 69.0 | 76.0 | 81.0 | 82.6 | 86.5 | 89.0 | -0.36 | 2.84 | 0.48 |
|  | NSF | Dry | 67 | 74.5 | 5.2 | 0.07 | 4.4 | 59.8 | 72.1 | 74.9 | 77.9 | 80.9 | 81.6 | 82.5 | 84.9 | -0.75 | 3.64 | <0.05 |
|  |  | Rainy | 70 | 75.2 | 6.7 | 0.09 | 6.4 | 57.0 | 71.2 | 75.0 | 79.8 | 83.9 | 84.7 | 87.2 | 88.2 | -0.35 | 2.94 | 0.46 |
|  | CCME1 | Dry | 67 | 88.8 | 17.4 | 0.20 | 0.0 | 41.1 | 87.8 | 100.0 | 100.0 | 100.0 | 100.0 | 100.0 | 100.0 | -1.69 | 4.64 | <0.05 |
|  |  | Rainy | 70 | 90.3 | 10.7 | 0.12 | 15.1 | 49.7 | 86.1 | 89.8 | 100.0 | 100.0 | 100.0 | 100.0 | 100.0 | -1.27 | 4.89 | <0.05 |
|  | CCME2 | Dry | 67 | 93.2 | 11.5 | 0.12 | 3.6 | 54.8 | 94.8 | 97.6 | 100.0 | 100.0 | 100.0 | 100.0 | 100.0 | -2.08 | 6.01 | <0.05 |
|  |  | Rainy | 70 | 95.4 | 5.6 | 0.06 | 3.9 | 75.0 | 93.5 | 97.4 | 100.0 | 100.0 | 100.0 | 100.0 | 100.0 | -1.90 | 6.97 | <0.05 |
| **PFA** | CETESB | Dry | 196 | 63.6 | 9.3 | 0.15 | 7.4 | 32.0 | 60.0 | 65.0 | 70.0 | 74.5 | 77.0 | 78.1 | 81.0 | -0.73 | 3.53 | <0.05 |
|  |  | Rainy | 199 | 67.2 | 9.7 | 0.14 | 8.9 | 34.0 | 62.0 | 69.0 | 74.0 | 77.0 | 79.1 | 83.0 | 91.0 | -0.78 | 3.83 | <0.05 |
|  | IGAM | Dry | 196 | 63.6 | 9.3 | 0.15 | 7.4 | 32.0 | 60.0 | 65.0 | 70.0 | 74.5 | 77.0 | 78.1 | 81.0 | -0.73 | 3.53 | <0.05 |
|  |  | Rainy | 199 | 67.2 | 9.7 | 0.14 | 8.9 | 34.0 | 62.0 | 69.0 | 74.0 | 77.0 | 79.1 | 83.0 | 91.0 | -0.78 | 3.83 | <0.05 |
|  | NSF | Dry | 196 | 71.4 | 5.6 | 0.08 | 5.8 | 55.4 | 67.6 | 71.6 | 75.4 | 77.8 | 79.9 | 81.7 | 88.2 | -0.14 | 2.95 | 0.42 |
|  |  | Rainy | 199 | 73.7 | 6.6 | 0.09 | 6.1 | 44.1 | 70.2 | 74.6 | 78.3 | 80.3 | 81.7 | 85.7 | 90.5 | -0.81 | 4.65 | <0.05 |
|  | CCME1 | Dry | 196 | 85.0 | 16.4 | 0.19 | 15.7 | 38.1 | 78.9 | 89.4 | 100.0 | 100.0 | 100.0 | 100.0 | 100.0 | -1.20 | 3.65 | <0.05 |
|  |  | Rainy | 199 | 88.2 | 15.2 | 0.17 | 15.1 | 33.5 | 80.8 | 89.8 | 100.0 | 100.0 | 100.0 | 100.0 | 100.0 | -1.63 | 5.32 | <0.05 |
|  | CCME2 | Dry | 196 | 88.2 | 10.6 | 0.12 | 5.3 | 42.9 | 87.0 | 91.4 | 94.7 | 97.3 | 98.2 | 100.0 | 100.0 | -2.00 | 6.80 | <0.05 |
|  |  | Rainy | 199 | 90.9 | 9.8 | 0.11 | 6.4 | 49.5 | 89.0 | 93.0 | 97.4 | 100.0 | 100.0 | 100.0 | 100.0 | -2.23 | 8.67 | <0.05 |
| **PAG** | CETESB | Dry | 299 | 60.4 | 9.9 | 0.16 | 10.4 | 32.0 | 54.0 | 62.0 | 68.0 | 72.0 | 74.0 | 76.0 | 78.0 | -0.62 | 2.78 | <0.05 |
|  |  | Rainy | 301 | 67.7 | 9.4 | 0.14 | 8.9 | 33.0 | 63.0 | 69.0 | 74.0 | 77.0 | 80.0 | 82.0 | 89.0 | -1.02 | 4.25 | <0.05 |
|  | IGAM | Dry | 299 | 60.4 | 9.9 | 0.16 | 10.4 | 32.0 | 54.0 | 62.0 | 68.0 | 72.0 | 74.0 | 76.0 | 78.0 | -0.62 | 2.78 | <0.05 |
|  |  | Rainy | 301 | 67.7 | 9.4 | 0.14 | 8.9 | 33.0 | 63.0 | 69.0 | 74.0 | 77.0 | 80.0 | 82.0 | 89.0 | -1.02 | 4.25 | <0.05 |
|  | NSF | Dry | 299 | 70.1 | 5.6 | 0.08 | 5.8 | 49.8 | 66.6 | 70.3 | 74.2 | 76.7 | 78.0 | 80.0 | 82.3 | -0.55 | 3.38 | <0.05 |
|  |  | Rainy | 301 | 75.0 | 6.9 | 0.09 | 5.7 | 45.6 | 71.6 | 75.5 | 79.3 | 82.8 | 84.7 | 86.3 | 93.6 | -0.89 | 5.09 | <0.05 |
|  | CCME1 | Dry | 299 | 78.3 | 19.2 | 0.25 | 21.5 | 29.3 | 67.4 | 83.6 | 89.8 | 100.0 | 100.0 | 100.0 | 100.0 | -0.73 | 2.42 | <0.05 |
|  |  | Rainy | 301 | 88.9 | 15.3 | 0.17 | 15.1 | 33.1 | 86.0 | 89.8 | 100.0 | 100.0 | 100.0 | 100.0 | 100.0 | -1.61 | 4.95 | <0.05 |
|  | CCME2 | Dry | 299 | 84.7 | 12.4 | 0.15 | 7.0 | 39.7 | 80.4 | 88.9 | 92.5 | 95.6 | 97.6 | 100.0 | 100.0 | -1.35 | 4.08 | <0.05 |
|  |  | Rainy | 301 | 91.2 | 9.9 | 0.11 | 4.4 | 41.6 | 89.7 | 94.6 | 97.5 | 100.0 | 100.0 | 100.0 | 100.0 | -2.41 | 9.50 | <0.05 |
| **MUN** | CETESB | Dry | 57 | 63.8 | 12.1 | 0.19 | 13.3 | 37.0 | 54.0 | 67.0 | 73.0 | 76.4 | 79.2 | 84.4 | 90.0 | -0.26 | 2.19 | 0.06 |
|  |  | Rainy | 59 | 69.8 | 11.4 | 0.16 | 8.9 | 39.0 | 65.5 | 71.0 | 76.5 | 81.4 | 88.0 | 88.8 | 93.0 | -0.70 | 3.59 | <0.05 |
|  | IGAM | Dry | 57 | 63.8 | 12.1 | 0.19 | 13.3 | 37.0 | 54.0 | 67.0 | 73.0 | 76.4 | 79.2 | 84.4 | 90.0 | -0.26 | 2.19 | 0.06 |
|  |  | Rainy | 59 | 69.8 | 11.4 | 0.16 | 8.9 | 39.0 | 65.5 | 71.0 | 76.5 | 81.4 | 88.0 | 88.8 | 93.0 | -0.70 | 3.59 | <0.05 |
|  | NSF | Dry | 57 | 73.5 | 6.6 | 0.09 | 5.6 | 57.8 | 70.2 | 74.8 | 78.1 | 80.4 | 82.3 | 86.1 | 88.2 | -0.36 | 2.74 | 0.19 |
|  |  | Rainy | 59 | 75.2 | 8.2 | 0.11 | 5.2 | 51.3 | 72.7 | 76.1 | 79.1 | 84.7 | 86.0 | 87.8 | 93.6 | -0.72 | 3.88 | <0.05 |
|  | CCME1 | Dry | 57 | 81.9 | 21.4 | 0.26 | 15.3 | 35.2 | 64.4 | 89.7 | 100.0 | 100.0 | 100.0 | 100.0 | 100.0 | -0.89 | 2.34 | <0.05 |
|  |  | Rainy | 59 | 88.7 | 17.3 | 0.20 | 0.0 | 40.9 | 88.4 | 100.0 | 100.0 | 100.0 | 100.0 | 100.0 | 100.0 | -1.67 | 4.64 | <0.05 |
|  | CCME2 | Dry | 57 | 86.4 | 15.8 | 0.18 | 8.4 | 44.3 | 80.5 | 91.9 | 97.6 | 100.0 | 100.0 | 100.0 | 100.0 | -1.52 | 4.33 | <0.05 |
|  |  | Rainy | 59 | 92.4 | 10.2 | 0.11 | 5.1 | 58.9 | 90.5 | 96.6 | 100.0 | 100.0 | 100.0 | 100.0 | 100.0 | -1.74 | 5.18 | <0.05 |
| **MIN** | CETESB | Dry | 17 | 61.7 | 9.6 | 0.16 | 11.9 | 46.0 | 56.0 | 60.0 | 70.0 | 72.2 | 75.2 | 78.1 | 80.0 | 0.13 | 2.13 | 0.93 |
|  |  | Rainy | 18 | 71.3 | 10.7 | 0.15 | 8.2 | 47.0 | 66.0 | 74.5 | 79.8 | 82.3 | 83.2 | 83.7 | 84.0 | -0.85 | 2.77 | 0.10 |
|  | IGAM | Dry | 17 | 61.7 | 9.6 | 0.16 | 11.9 | 46.0 | 56.0 | 60.0 | 70.0 | 72.2 | 75.2 | 78.1 | 80.0 | 0.13 | 2.13 | 0.93 |
|  |  | Rainy | 18 | 71.3 | 10.7 | 0.15 | 8.2 | 47.0 | 66.0 | 74.5 | 79.8 | 82.3 | 83.2 | 83.7 | 84.0 | -0.85 | 2.77 | 0.10 |
|  | NSF | Dry | 17 | 70.4 | 5.1 | 0.07 | 5.8 | 63.7 | 66.8 | 70.7 | 73.6 | 76.3 | 78.0 | 80.2 | 81.6 | 0.49 | 2.45 | 0.47 |
|  |  | Rainy | 18 | 76.5 | 8.0 | 0.10 | 8.3 | 59.6 | 73.0 | 76.3 | 83.6 | 84.6 | 84.8 | 85.6 | 86.1 | -0.83 | 2.90 | 0.05 |
|  | CCME1 | Dry | 17 | 81.4 | 18.2 | 0.22 | 15.7 | 36.8 | 72.6 | 89.4 | 89.8 | 100.0 | 100.0 | 100.0 | 100.0 | -1.02 | 3.23 | <0.05 |
|  |  | Rainy | 18 | 90.8 | 12.4 | 0.14 | 7.6 | 55.8 | 89.5 | 94.9 | 100.0 | 100.0 | 100.0 | 100.0 | 100.0 | -1.43 | 4.48 | <0.05 |
|  | CCME2 | Dry | 17 | 84.7 | 13.3 | 0.16 | 7.7 | 49.2 | 82.8 | 88.0 | 93.1 | 96.2 | 97.0 | 98.8 | 100.0 | -1.38 | 4.25 | <0.05 |
|  |  | Rainy | 18 | 91.8 | 6.7 | 0.07 | 5.6 | 75.9 | 90.0 | 93.4 | 97.3 | 97.6 | 98.0 | 99.2 | 100.0 | -1.05 | 3.27 | <0.05 |
| **PER** | CETESB | Dry | 83 | 54.0 | 11.8 | 0.22 | 13.3 | 23.0 | 47.0 | 54.0 | 63.5 | 69.0 | 72.9 | 74.4 | 80.0 | -0.06 | 2.46 | 0.50 |
|  |  | Rainy | 82 | 59.3 | 12.5 | 0.21 | 10.4 | 24.0 | 53.0 | 61.0 | 68.0 | 73.0 | 76.9 | 78.4 | 83.0 | -0.79 | 3.52 | <0.05 |
|  | IGAM | Dry | 83 | 54.0 | 11.8 | 0.22 | 13.3 | 23.0 | 47.0 | 54.0 | 63.5 | 69.0 | 72.9 | 74.4 | 80.0 | -0.06 | 2.46 | 0.50 |
|  |  | Rainy | 82 | 59.3 | 12.5 | 0.21 | 10.4 | 24.0 | 53.0 | 61.0 | 68.0 | 73.0 | 76.9 | 78.4 | 83.0 | -0.79 | 3.52 | <0.05 |
|  | NSF | Dry | 83 | 66.5 | 7.8 | 0.12 | 7.6 | 39.8 | 62.7 | 67.2 | 72.4 | 75.0 | 77.1 | 79.1 | 81.1 | -0.85 | 4.03 | <0.05 |
|  |  | Rainy | 82 | 69.0 | 10.6 | 0.15 | 9.1 | 36.2 | 63.0 | 71.5 | 76.5 | 79.6 | 81.9 | 85.7 | 89.4 | -0.82 | 3.62 | <0.05 |
|  | CCME1 | Dry | 83 | 64.7 | 23.1 | 0.36 | 32.3 | 24.0 | 42.2 | 67.8 | 86.0 | 100.0 | 100.0 | 100.0 | 100.0 | 0.02 | 1.65 | <0.05 |
|  |  | Rainy | 82 | 74.1 | 21.4 | 0.29 | 27.3 | 23.2 | 56.6 | 79.8 | 89.7 | 100.0 | 100.0 | 100.0 | 100.0 | -0.55 | 2.22 | <0.05 |
|  | CCME2 | Dry | 83 | 74.7 | 18.3 | 0.24 | 20.4 | 42.0 | 57.9 | 80.0 | 90.1 | 95.3 | 97.5 | 97.6 | 97.6 | -0.42 | 1.73 | <0.05 |
|  |  | Rainy | 82 | 85.2 | 12.7 | 0.15 | 8.1 | 50.5 | 77.8 | 89.7 | 94.4 | 97.2 | 97.6 | 100.0 | 100.0 | -1.16 | 3.39 | <0.05 |
| **MUX** | CETESB | Dry | 30 | 51.9 | 13.9 | 0.27 | 18.5 | 25.0 | 44.3 | 49.0 | 63.0 | 68.3 | 72.1 | 75.1 | 78.0 | 0.00 | 2.14 | 0.49 |
|  |  | Rainy | 30 | 58.2 | 18.3 | 0.31 | 15.6 | 16.0 | 46.3 | 63.0 | 71.8 | 77.1 | 80.8 | 83.0 | 83.0 | -0.73 | 2.55 | <0.05 |
|  | IGAM | Dry | 30 | 51.9 | 13.9 | 0.27 | 18.5 | 25.0 | 44.3 | 49.0 | 63.0 | 68.3 | 72.1 | 75.1 | 78.0 | 0.00 | 2.14 | 0.49 |
|  |  | Rainy | 30 | 58.2 | 18.3 | 0.31 | 15.6 | 16.0 | 46.3 | 63.0 | 71.8 | 77.1 | 80.8 | 83.0 | 83.0 | -0.73 | 2.55 | <0.05 |
|  | NSF | Dry | 30 | 64.9 | 9.4 | 0.14 | 10.1 | 44.7 | 59.2 | 65.8 | 72.1 | 74.1 | 75.4 | 77.5 | 79.9 | -0.74 | 2.73 | <0.05 |
|  |  | Rainy | 30 | 65.8 | 15.0 | 0.23 | 14.8 | 32.9 | 56.1 | 69.1 | 77.0 | 82.4 | 83.7 | 85.6 | 87.7 | -0.59 | 2.27 | 0.06 |
|  | CCME1 | Dry | 30 | 60.4 | 25.2 | 0.42 | 33.6 | 16.3 | 41.1 | 55.0 | 83.8 | 90.8 | 100.0 | 100.0 | 100.0 | 0.10 | 1.72 | 0.09 |
|  |  | Rainy | 30 | 72.5 | 26.2 | 0.36 | 22.5 | 19.6 | 57.6 | 80.4 | 89.8 | 100.0 | 100.0 | 100.0 | 100.0 | -0.77 | 2.26 | <0.05 |
|  | CCME2 | Dry | 30 | 72.3 | 18.7 | 0.26 | 24.8 | 41.7 | 53.3 | 74.4 | 90.0 | 92.5 | 95.6 | 98.3 | 100.0 | -0.17 | 1.54 | <0.05 |
|  |  | Rainy | 30 | 81.1 | 18.0 | 0.22 | 10.2 | 39.3 | 70.5 | 90.7 | 92.9 | 97.6 | 97.6 | 98.6 | 100.0 | -1.00 | 2.72 | <0.05 |
| **LUM** | CETESB | Dry | 38 | 50.5 | 12.4 | 0.24 | 11.1 | 24.0 | 42.0 | 49.0 | 57.5 | 68.9 | 73.2 | 74.0 | 74.0 | 0.21 | 2.63 | 0.35 |
|  |  | Rainy | 38 | 53.5 | 15.4 | 0.29 | 19.3 | 22.0 | 40.8 | 56.5 | 64.0 | 73.3 | 74.3 | 76.3 | 77.0 | -0.31 | 2.15 | 0.14 |
|  | IGAM | Dry | 38 | 50.5 | 12.4 | 0.24 | 11.1 | 24.0 | 42.0 | 49.0 | 57.5 | 68.9 | 73.2 | 74.0 | 74.0 | 0.21 | 2.63 | 0.35 |
|  |  | Rainy | 38 | 53.5 | 15.4 | 0.29 | 19.3 | 22.0 | 40.8 | 56.5 | 64.0 | 73.3 | 74.3 | 76.3 | 77.0 | -0.31 | 2.15 | 0.14 |
|  | NSF | Dry | 38 | 63.0 | 7.7 | 0.12 | 6.5 | 44.5 | 58.9 | 62.2 | 68.1 | 74.5 | 75.7 | 76.0 | 76.0 | -0.17 | 2.94 | 0.27 |
|  |  | Rainy | 38 | 65.3 | 12.4 | 0.19 | 15.4 | 40.4 | 56.6 | 66.2 | 76.6 | 80.6 | 82.0 | 84.3 | 86.7 | -0.21 | 2.02 | 0.31 |
|  | CCME1 | Dry | 38 | 56.8 | 22.9 | 0.40 | 24.5 | 29.8 | 38.2 | 50.7 | 76.6 | 92.9 | 100.0 | 100.0 | 100.0 | 0.61 | 2.10 | <0.05 |
|  |  | Rainy | 38 | 66.6 | 22.3 | 0.34 | 29.3 | 29.7 | 47.7 | 67.5 | 85.2 | 100.0 | 100.0 | 100.0 | 100.0 | -0.03 | 1.73 | <0.05 |
|  | CCME2 | Dry | 38 | 70.7 | 18.3 | 0.26 | 27.5 | 41.6 | 54.6 | 68.6 | 87.7 | 92.8 | 95.0 | 97.6 | 100.0 | -0.03 | 1.54 | <0.05 |
|  |  | Rainy | 38 | 81.4 | 14.5 | 0.18 | 14.9 | 53.4 | 73.2 | 85.6 | 92.5 | 97.0 | 100.0 | 100.0 | 100.0 | -0.52 | 1.99 | <0.05 |
| **URB** | CETESB | Dry | 48 | 42.4 | 11.1 | 0.26 | 8.9 | 18.0 | 35.8 | 41.0 | 48.0 | 54.6 | 64.7 | 66.3 | 71.0 | 0.39 | 3.39 | 0.33 |
|  |  | Rainy | 48 | 47.0 | 15.5 | 0.33 | 19.3 | 15.0 | 33.8 | 47.0 | 57.3 | 67.6 | 76.0 | 78.0 | 78.0 | 0.23 | 2.42 | 0.41 |
|  | IGAM | Dry | 48 | 42.4 | 11.1 | 0.26 | 8.9 | 18.0 | 35.8 | 41.0 | 48.0 | 54.6 | 64.7 | 66.3 | 71.0 | 0.39 | 3.39 | 0.33 |
|  |  | Rainy | 48 | 47.0 | 15.5 | 0.33 | 19.3 | 15.0 | 33.8 | 47.0 | 57.3 | 67.6 | 76.0 | 78.0 | 78.0 | 0.23 | 2.42 | 0.41 |
|  | NSF | Dry | 48 | 57.0 | 9.0 | 0.16 | 8.3 | 36.2 | 48.2 | 59.1 | 63.0 | 66.4 | 70.8 | 72.6 | 73.2 | -0.36 | 2.40 | 0.15 |
|  |  | Rainy | 48 | 62.2 | 14.4 | 0.23 | 14.3 | 26.6 | 51.6 | 60.2 | 70.2 | 83.1 | 87.0 | 90.3 | 90.5 | 0.17 | 2.62 | 0.40 |
|  | CCME1 | Dry | 48 | 43.6 | 19.6 | 0.45 | 10.6 | 23.3 | 30.2 | 36.8 | 45.2 | 74.9 | 89.6 | 100.0 | 100.0 | 1.74 | 5.11 | <0.05 |
|  |  | Rainy | 48 | 54.0 | 22.3 | 0.41 | 20.5 | 19.0 | 36.2 | 47.8 | 65.2 | 89.7 | 100.0 | 100.0 | 100.0 | 0.75 | 2.52 | <0.05 |
|  | CCME2 | Dry | 48 | 58.5 | 18.0 | 0.31 | 14.3 | 39.6 | 42.9 | 52.3 | 70.5 | 88.4 | 93.9 | 95.1 | 95.4 | 0.86 | 2.34 | <0.05 |
|  |  | Rainy | 48 | 73.0 | 16.0 | 0.22 | 21.8 | 45.5 | 59.2 | 73.7 | 86.4 | 95.1 | 97.1 | 97.6 | 97.6 | -0.04 | 1.77 | <0.05 |

^a^ **LULC classes**: BPA = Better preserved areas; PFA = Pasture, forestry and agriculture; PAG = Pasture and agriculture; MUN = Multi-use with significant natural cover and mining; MIN = Major mining complexes; PER = Peri-urban areas; MUX = Mixed urban use; LUM = Large urban centers and mining; URB = Major urban centers.

^b^ **WQI methods**:

**CETESB** – Companhia de Tecnologia de Saneamento Ambiental, 2005. Relatório de águas interiores do Estado de São Paulo de 2005. Available in: <http://www.cetesb. sp.gov.br/Agua> Accessed on 02 jun. 2025.

**IGAM** - Instituto Mineiro de Gestão das Águas, 2018. Índice de Qualidade Das Águas – IQA. Available in: <https://igam.mg.gov.br/w/indice-de-qualidade-das-aguas-iqa>. Accessed on 04 jan 2025.

**NSF** - Brown, R. M., McClelland, N. I., Deininger, R. A., & Tozer, R. G. (1970). A water quality index-do we dare. Water and sewage works, 117(10).

**CCME** - Canadian Council of Ministers of the Environment, 2017. Canadian Water Quality Guidelines for the Protection of Aquatic Life. CCME water quality index user’s manual 2017 update. Available in: <https://ccme.ca/en/resources/water-quality> Accessed on 14 oct. 2025.

Note: CCME1 considered the same parameters from CETESB and CCME2 considered selected parameters contemplated in National Environment Council of Brazil (CONAMA, 2005).

**CONAMA** - Conselho Nacional do Meio Ambiente, 2005. Resolução nº 357, de 17 de março de 2005. Dispõe sobre a classificação dos corpos de água e diretrizes ambientais para o seu enquadramento, bem como estabelece as condições e padrões de lançamento de efluentes, e dá outras providências. Diário Oficial da União, Brasília, DF, n. 53, 18 mar. 2005, Seção 1, pp. 58–63.

**Statistical metrics:** n = number of samples; x̅ = Mean; σ = standard deviation; CV = Coefficient of variation; Min = Minimum; P = Percentile values (25^th^, 50^th^ = median, 75^th^, 90^th^, 95^th^, and 98^th^); Max = Maximum; Skew = Skewness; Rku = Kurtosis; pSW = p-value of the Shapiro-Wilk normality test.


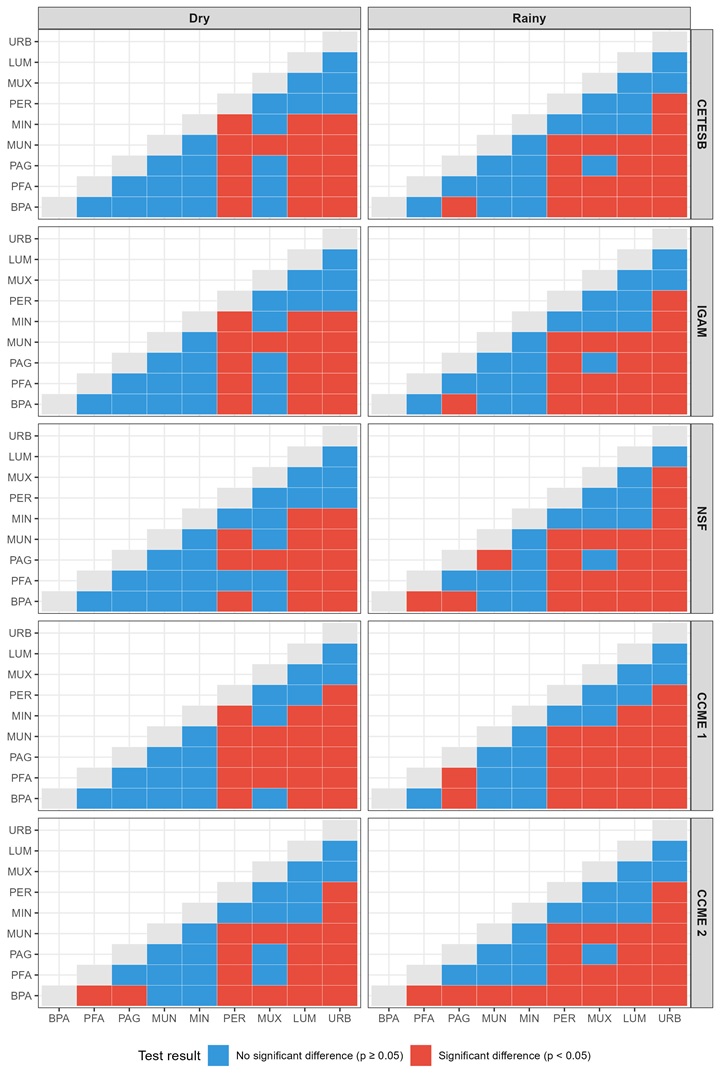


# **Appendix C** - Results of Dunn’s test for pairwise comparisons among land use and land cover (LULC) classes, considering different water quality index (WQI) methodologies. and seasonal conditions (dry and rainy periods). Red cells indicate statistically significant differences (ρ < 0.05), while blue cells indicate no significant differences (ρ ≥ 0.05). LULC classes: BPA = Better preserved areas; PFA = Pasture, forestry and agriculture; PAG = Pasture and agriculture; MUN = Multi-use with significant natural cover and mining; MIN = Major mining complexes; PER = Peri-urban areas; MUX = Mixed urban use; LUM = Large urban centers and mining; URB = Major urban centers.
